# Predicting COVID-19 Severity with a Specific Nucleocapsid Antibody plus Disease Risk Factor Score

**DOI:** 10.1128/mSphere.00203-21

**Published:** 2021-04-28

**Authors:** Sanjana R. Sen, Emily C. Sanders, Kristin N. Gabriel, Brian M. Miller, Hariny M. Isoda, Gabriela S. Salcedo, Jason E. Garrido, Rebekah P. Dyer, Rie Nakajima, Aarti Jain, Ana-Maria Caldaruse, Alicia M. Santos, Keertna Bhuvan, Delia F. Tifrea, Joni L. Ricks-Oddie, Philip L. Felgner, Robert A. Edwards, Sudipta Majumdar, Gregory A. Weiss

**Affiliations:** a Department of Molecular Biology & Biochemistry, University of California Irvine, Irvine, California, USA; b Department of Chemistry, University of California Irvine, Irvine, California, USA; c Department of Physiology and Biophysics, University of California Irvine, Irvine, California, USA; d Department of Pharmaceutical Sciences, University of California Irvine, Irvine, California, USA; e Department of Pathology and Laboratory Medicine, University of California Irvine, Irvine, California, USA; f Center for Statistical Consulting, Department of Statistics, University of California Irvine, Irvine, California, USA; g Biostatics, Epidemiology and Research Design Unit, Institute for Clinical and Translational Sciences, University of California Irvine, Irvine, California, USA; University of Maryland School of Medicine

**Keywords:** SARS-CoV-2, coronaviruses, epitope mapping, phage display, prognostic

## Abstract

Effective methods for predicting COVID-19 disease trajectories are urgently needed. Here, enzyme-linked immunosorbent assay (ELISA) and coronavirus antigen microarray (COVAM) analysis mapped antibody epitopes in the plasma of COVID-19 patients (*n* = 86) experiencing a wide range of disease states. The experiments identified antibodies to a 21-residue epitope from nucleocapsid (termed Ep9) associated with severe disease, including admission to the intensive care unit (ICU), requirement for ventilators, or death. Importantly, anti-Ep9 antibodies can be detected within 6 days post-symptom onset and sometimes within 1 day. Furthermore, anti-Ep9 antibodies correlate with various comorbidities and hallmarks of immune hyperactivity. We introduce a simple-to-calculate, disease risk factor score to quantitate each patient’s comorbidities and age. For patients with anti-Ep9 antibodies, scores above 3.0 predict more severe disease outcomes with a 13.42 likelihood ratio (96.7% specificity). The results lay the groundwork for a new type of COVID-19 prognostic to allow early identification and triage of high-risk patients. Such information could guide more effective therapeutic intervention.

**IMPORTANCE** The COVID-19 pandemic has resulted in over two million deaths worldwide. Despite efforts to fight the virus, the disease continues to overwhelm hospitals with severely ill patients. Diagnosis of COVID-19 is readily accomplished through a multitude of reliable testing platforms; however, prognostic prediction remains elusive. To this end, we identified a short epitope from the SARS-CoV-2 nucleocapsid protein and also a disease risk factor score based upon comorbidities and age. The presence of antibodies specifically binding to this epitope plus a score cutoff can predict severe COVID-19 outcomes with 96.7% specificity.

## Abstract (precis)

The COVID-19 pandemic has resulted in over two million deaths worldwide. Despite efforts to fight the virus, the disease continues to overwhelm hospitals with severely ill patients.

## INTRODUCTION

The COVID-19 pandemic has triggered an ongoing global health crisis. More than 119.8 million confirmed cases and 2.7 million deaths have been reported worldwide as of 16 March 2021 (1). The virus that causes COVID-19, severe acute respiratory syndrome coronavirus (SARS-CoV-2), belongs to the same family of viruses responsible for respiratory illness linked to recent epidemics—severe acute respiratory syndrome (SARS-CoV-1, termed SARS here) in 2002 to 2003 and Middle East respiratory syndrome (MERS) in 2012 (2). The current and previous outbreaks suggest coronaviruses will remain viruses of concern for global health.

Many risk factors and comorbidities, including age, sex, hypertension, diabetes, and obesity, can influence COVID-19 patient outcomes (3). Analysis of patient immune parameters has linked disease severity to elevated levels of biomarkers for inflammation (C-reactive protein [CRP] and cardiac troponin I), organ damage (aspartate aminotransferase [AST] and hypoalbuminemia), immune hyperactivity (interleukin-6 [IL-6] and IL-10), and clotting (d-dimer) (4). Mortality in COVID-19 is often caused by multiorgan injury and severe pneumonia attributed to an excessive immune response, termed a cytokine storm (5). Given the rapid and wide spectrum of COVID-19 disease progression, a more precise prognostic linking disease risk factors and specific immune responses can potentially predict disease trajectories and guide interventions.

One hypothesis to explain differences in severity of COVID-19 implicates weakly binding, nonneutralizing antibodies (Abs) to SARS-CoV-2 proteins (6). However, the potential harm of these suboptimal Abs in COVID-19 patient outcomes remains ill defined. Furthermore, a recent review on antibody-dependent enhancement of SARS-CoV-2 stated, “At present, there are no known clinical findings, immunological assays or biomarkers that can differentiate any severe infection from immune-enhanced disease, whether by measuring antibodies, T cells or intrinsic host responses” (7). This conclusion inspired our study.

SARS-CoV-2 encodes four major structural proteins—spike (S), nucleocapsid (N), membrane (M), and envelope (E). The S, N, and M proteins from SARS elicit an Ab-based immune response (8, 9). The Ab response and its effects on disease progression in SARS-CoV-2 remain under investigation (10, 11). Bioinformatics has predicted >55 Ab binding epitope regions from SARS-CoV-2 (12–17). The epitopes for N, M, or E proteins are less well-characterized than those for S protein. Several studies have reported comprehensive epitope mapping of the antibody response to SARS-CoV-2 (18–21). Here, we sought to characterize epitopes from SARS-CoV-2 and their correlations with disease severity. Enzyme-linked immunosorbent assays (ELISAs) with phage-displayed epitopes (phage ELISAs) and coronavirus antigen microarray (COVAM) analysis (22) examined plasma samples from COVID-19 patients (*n* = 86). The results demonstrate that Abs to a specific epitope from N protein plus disease risk factors strongly correlate with COVID-19 disease severity.

(This article was submitted to an online preprint archive [23].)

## RESULTS

### Design and production of candidate epitopes.

**FIG 1:**
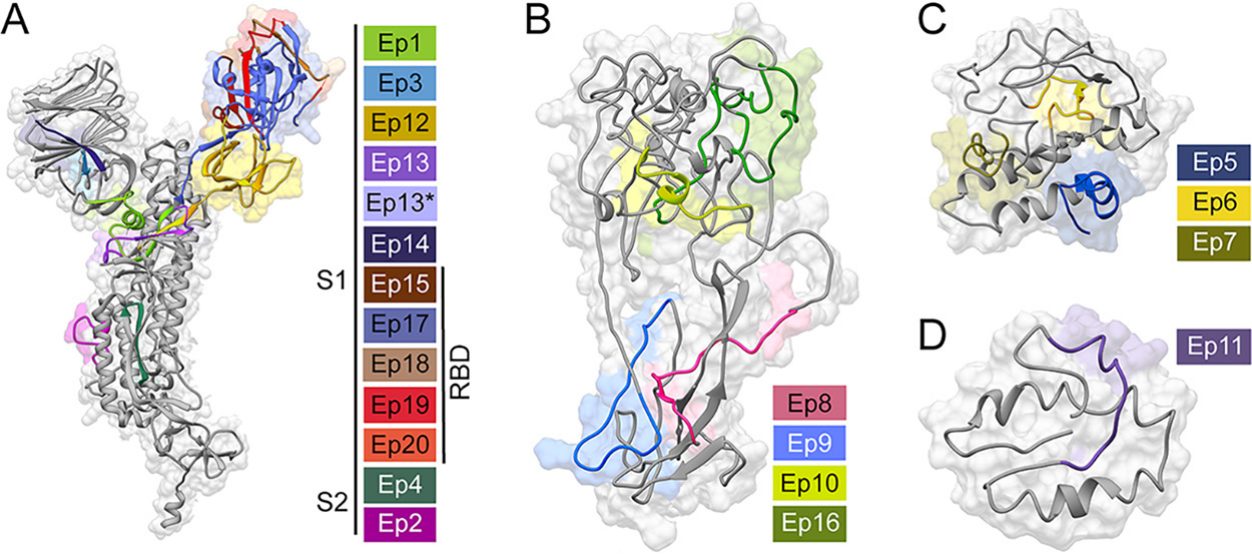
Predicted SARS-CoV-2 epitopes examined by phage ELISA. Structural models (gray) of the SARS-CoV-2 S (A), N (B), M (C), or E (D) proteins illustrate our epitope design (colored). Sequence Ep13* has the mutation D614G, which increases the fitness of SARS-CoV-2 (51–53). The depicted structural models were derived from an S protein X-ray structure (PDB: 6VXX) (24) or computation modeling of N, M, and E proteins (Protein Gene Bank: QHD43423, QHD43419, and QHD43418, respectively) (54). Table S1 provides sequences, sources, and rationale for epitope design.

Twenty-one putative SARS-CoV-2 epitopes were predicted through bioinformatics (12–14) and structure-based analysis. The candidate epitopes spanned the S, N, M, or E proteins and were on average 34 amino acids in length (Fig. 1 and also Table S1 in the supplemental material). These epitopes were phage-displayed as fragments of the full-length protein and were likely unstructured. Here, epitope refers to the predicted region of the antigenic protein recognized by the antibody’s paratope. The structure of S protein bound to a neutralizing antibody (24, 25) provided the starting point for 12 of these antibody epitopes. Epitopes were designed to potentially isolate even suboptimal Abs binding to small portions of these structural proteins; such suboptimal Abs were hypothesized to provide insight into disease severity. After display of each potential epitope on the surface of phage, the quality of the epitopes was evaluated by PCR, DNA sequencing, and quality control (QC) ELISA (Fig. S1). A total of 18 phage-displayed, putative epitopes passed quality control PCR and were selected for further study.

10.1128/mSphere.00203-21.1 FIG S1 Quality control ELISA (QC ELISA) for phage-displayed epitope candidates. Anti-FLAG antibodies (1:1,000 in coating buffer) were immobilized on a microtiter plate. Subsequent steps followed the ELISA protocol provided here. Error bars represent SEM (*n* = 3). Ep8 was apparently toxic to E. coli, and Ep7 and Ep19 repeatedly failed sequencing quality controls after phage propagation. Download FIG S1, PDF file, 0.1 MB. Copyright © 2021 Sen et al. 2021 Sen et al. https://creativecommons.org/licenses/by/4.0/ This content is distributed under the terms of the Creative Commons Attribution 4.0 International license.

10.1128/mSphere.00203-21.8 TABLE S1 Phage-displayed putative epitopes of SARS-CoV-2 and Ep9 orthologous sequences from SARS, MERS, HKU-1, and NL63. Download Table S1, PDF file, 0.6 MB. Copyright © 2021 Sen et al. 2021 Sen et al. https://creativecommons.org/licenses/by/4.0/ This content is distributed under the terms of the Creative Commons Attribution 4.0 International license.

### Mapping epitope binding to anti-SARS-CoV-2 Abs.

**FIG 2:**
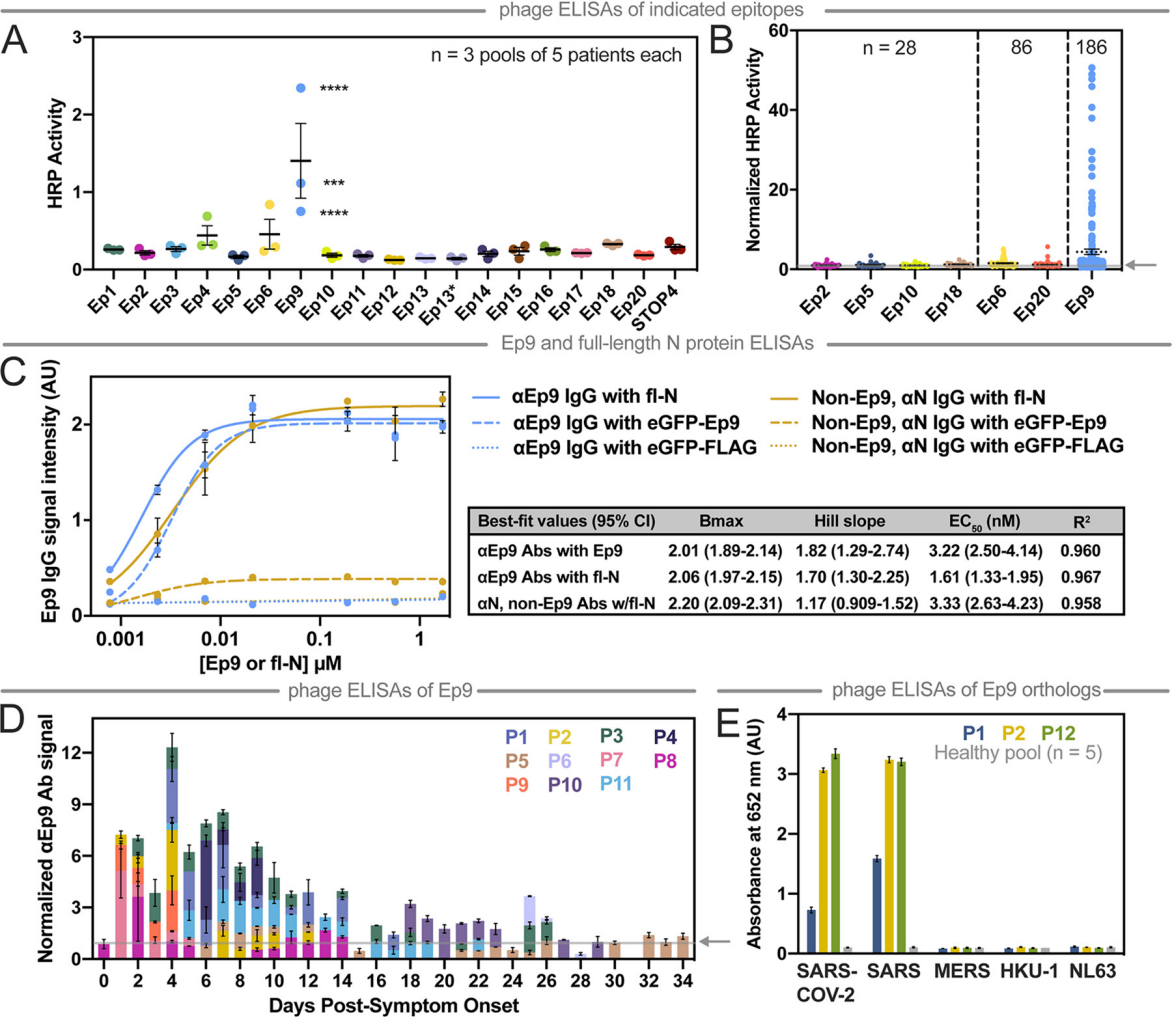
Mapping COVID-19 patient antibody responses with phage-displayed SARS-CoV-2 epitopes. (A) This phage ELISA with the indicated epitopes (*x* axis) examined plasma pooled from patients (*n* = 3 pools of 5 patients each, 2 technical replicates). STOP4 is the phage negative control. (B) The epitopes with the highest signals were then further examined by ELISA with plasma from individual patients (*n* as indicated). (C) This ELISA measures dose-dependent binding of αN IgGs from plasma pooled from five αEp9-positive patients and five non-Ep9, αN-positive patients to eGFP-Ep9 (dashed line), eGFP negative control (eGFP-FLAG, dotted line), or full-length N protein (fl-N, solid line). The indicated concentrations of Ep9 or fl-N were immobilized on microtiter plates, and binding of pooled patient plasma (1:100) was detected using α-Fc IgG-HRP Abs (1:10,000). Pooled patients were matched by similar αN IgG binding signal in COVAM analysis (inset). Nonlinear lines of best fit for binding saturation are represented. Statistical comparisons of *B*_max_, Hill slope, and EC_50_ between groups determines that binding of αEp9 IgGs to fl-N or eGFP-Ep9 and that of non-Ep9, αN IgGs to fl-N are significantly different (*P* < 0.0001). Error bars represent ±SD. The data demonstrate that the EC_50_ value of αEp9 Abs is equal to the cumulative EC_50_ of all other αN Abs in patients lacking the αEp9 Abs. In the presence of the αEp9 Abs, the apparent binding levels of αN Abs against fl-N approximately double. (D) With samples from individual patients (designated P# and by color) collected at the indicated times, αEp9 Abs were measured. The subset of patients shown here comprises all samples for which longitudinal data were available. (E) Phage ELISA with samples from patients with strong αEp9 Ab responses (two from the longitudinal study and one from the patient population) examines cross-reactive binding to Ep9 or Ep9 orthologs from the indicated coronaviruses (*x* axis, 3 technical replicates). The arrow on the *y* axis and gray line (B and D) represents the negative control used for normalizing the data. Error bars represent SEM (A, B, C, and E) or range of two measurements (D).

Plasma from COVID-19 patients was subjected to ELISAs with the phage-displayed SARS-CoV-2 epitopes (Fig. 2A). Unless otherwise indicated (e.g., healthy controls), plasma refers to samples from PCR-verified, COVID-19 patients. In this initial assay, plasma was pooled, diluted 100-fold, and applied as a coating on a microtiter plate as the target antigen for binding to the phage-displayed epitopes (3 pools of *n* = 5 patients per pool). Nonspecific interactions were blocked (ChonBlock), and phage-displayed epitopes were added for ELISA. The resultant data were normalized by signal from the corresponding negative control (phage without a displayed epitope). Seven candidate epitopes from the pooled patients were further investigated with a larger number of individual patient samples (*n* = 28) (Fig. 2B). The strongest reproducible binding was observed for three epitopes from M (Ep6), N (Ep9), and S (Ep20) proteins. Additional COVID-19 plasma samples were profiled for binding to these three epitopes (*n* = 86 total) (Fig. 2B).

Only the Ep9 epitope from N protein demonstrated robust, statistically significant antibody binding in 27% of patients (*n* = 186) (Fig. 2B). Of these patients, 100 did not have corresponding health information and were not analyzed further in this report. To test non-phage-displayed epitopes, dose-dependent binding of antibodies to Ep9 fused to enhanced green fluorescent protein (eGFP-Ep9) or to full-length N protein demonstrated that anti-Ep9 (αEp9) IgGs bound its antigen with a 50% effective concentration (EC_50_) of 3.22 nM (95% confidence interval [CI] = 2.49 to 4.14 nM). This experiment examined plasma samples with the highest IgG response against the N protein in the COVAM assay. Patients without αEp9 Abs had roughly the same level of binding to N protein as observed for αEp9 Abs binding to Ep9. However, such αEp9 Abs appeared to add to N protein binding by antibodies; an approximately 2-fold increase in apparent antibody binding levels for N protein was observed, if the patient also had αEp9 Abs (Fig. 2C). In patients for whom longitudinal samples were available, the highest levels of αEp9 Abs were observed at days 1 to 14 post-symptom onset (*n* = 11) and were detectable within 6 days (Fig. 2D). In four of these patients, αEp9 Abs persisted after day 14.

### Cross-reactivity of αEp9 Abs against orthologous epitopes from other coronaviruses.

Next, the cross-reactivity of αEp9 Abs was examined with Ep9 orthologs from four phylogenetically related coronaviruses known to infect humans (Fig. S2A). Specifically, plasma with αEp9 Abs (*n* = 3) and pooled plasma from healthy individuals (*n* = 5) were assayed. The Ep9 epitopes from SARS-CoV-2 and SARS have 90% amino acid sequence homology. Unsurprisingly, this high degree of similarity resulted in a cross-reactive Ep9 epitope, and a strong antibody response was observed to Ep9 epitopes from both viruses (Fig. 2E). The coronaviruses MERS, HKU-1, and NL63 have 52%, 43%, and 8% sequence homology to SARS-CoV-2 Ep9, respectively (Fig. S2B). These more distantly related orthologs exhibited no cross-reactivity with the αEp9 Abs. Furthermore, no response was observed to Ep9 in pooled plasma from healthy individuals.

10.1128/mSphere.00203-21.2 FIG S2 Epitope homology of SARS-CoV-2 with four phylogenetically related coronaviruses known to infect humans. (A) Evolutionary lineages of the human coronaviruses investigated here, including the highly pathogenic (SARS-CoV-2, SARS, and MERS) and the less virulent (HKU-1 and NL63). (B) The pairwise homology (% amino acid identity) between SARS-CoV-2 and the indicated coronavirus. Labels (top) indicate the proteins and domains (e.g., S1) from which the epitopes are derived. Download FIG S2, PDF file, 0.2 MB. Copyright © 2021 Sen et al. 2021 Sen et al. https://creativecommons.org/licenses/by/4.0/ This content is distributed under the terms of the Creative Commons Attribution 4.0 International license.

The protein microarray COVAM analysis is a high-throughput serological test for SARS-CoV-2 Ab cross-reactivity with a panel of 61 antigens from 23 strains of 10 respiratory tract infection-causing viruses (22). In this assay, each antigen was printed onto microarrays, probed with human plasma, and analyzed with an ArrayCam imager. COVAM distinguishes between IgG and IgM Abs binding to the full-length N protein (Fig. S3 and S4, respectively). Thus, the COVAM analysis complemented the phage ELISA by expanding the scope of antigens surveyed and adding Ab serotype information. The ELISA and COVAM data both demonstrated that αEp9 Abs were highly specific for lineage B betacoronaviruses and were unlikely to be found in patients before their infection with SARS-CoV-2.

10.1128/mSphere.00203-21.3 FIG S3 COVAM data showing the variation in IgG seroreactivity of patient plasma. The heatmap shows normalized signal intensity from plasma samples (*n* = 45). Plasma samples are in columns and sorted left to right by increasing average intensity to differentially reactive IgG, and viruses are in rows sorted by decreasing average seroreactivity. Download FIG S3, PDF file, 0.4 MB. Copyright © 2021 Sen et al. 2021 Sen et al. https://creativecommons.org/licenses/by/4.0/ This content is distributed under the terms of the Creative Commons Attribution 4.0 International license.

10.1128/mSphere.00203-21.4 FIG S4 Variation in IgM seroreactivity of patient plasma. Heatmap showing normalized signal intensity from plasma samples (*n* = 45). Plasma samples are in columns and sorted left to right by increasing average intensity to differentially reactive IgM, and viruses are in rows sorted by decreasing average seroreactivity. Download FIG S4, PDF file, 0.4 MB. Copyright © 2021 Sen et al. 2021 Sen et al. https://creativecommons.org/licenses/by/4.0/ This content is distributed under the terms of the Creative Commons Attribution 4.0 International license.

### More severe disease and poorer outcomes for αEp9 patients.

**FIG 3:**
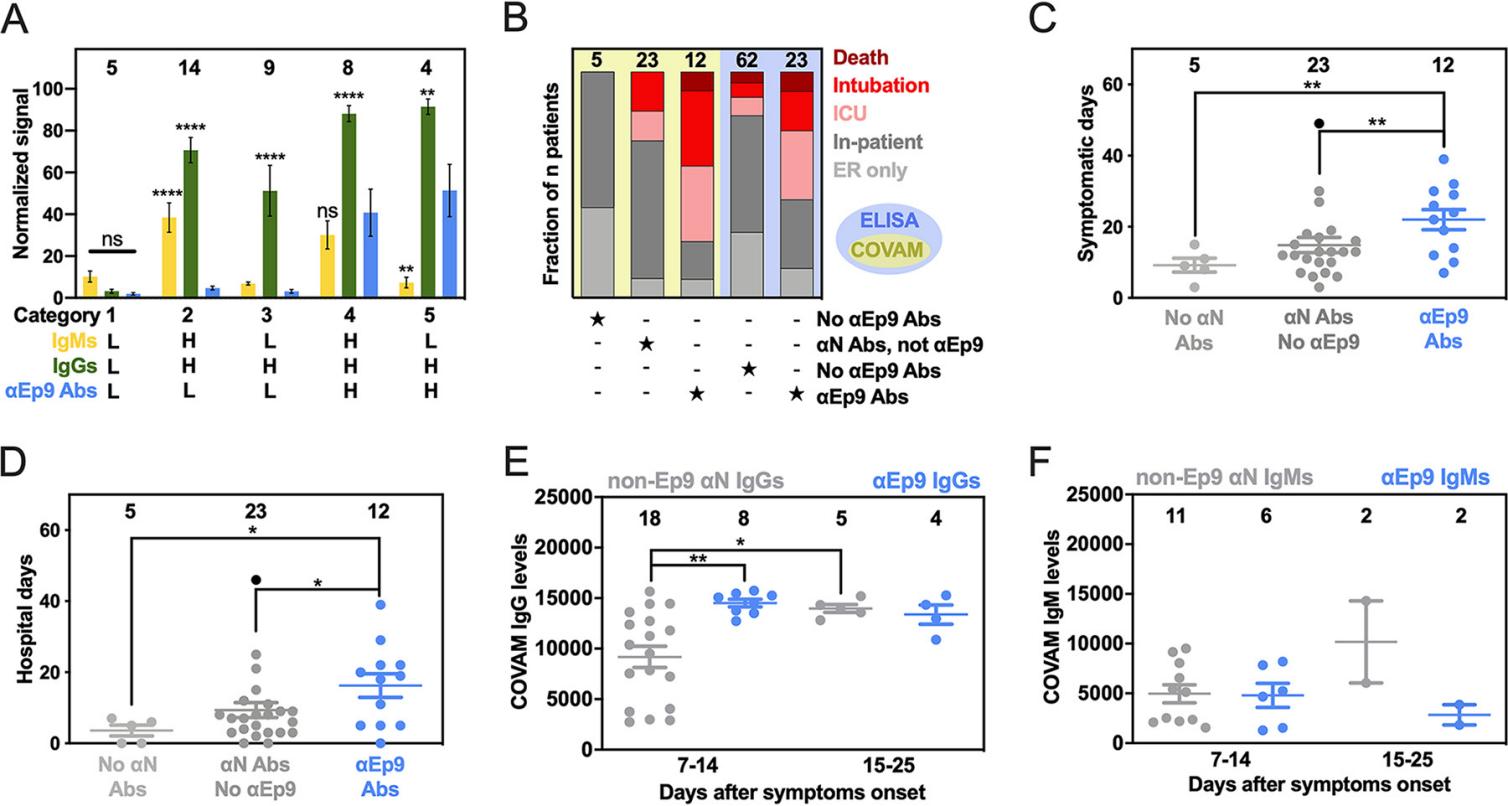
Patients with αEp9 Abs have more severe disease. (A) Normalized and categorized data from measurements by COVAM (IgMs in yellow, IgGs in green) and Ep9 phage ELISA (blue). ANOVA comparing COVAM to ELISA with Dunnett’s multiple-comparison test yields *P* values of <0.01 (**), <0.0001 (****), or not significant (ns). (B) Disease severity (color) binned by antibody response (filled star) for COVAM (yellow) or ELISA (blue). Statistical analysis reveals significant differences between distributions of severe and nonsevere disease comparing patient categories, *P* < 0.01 (Chi-squared test) and *P* < 0.001 (Fisher’s exact test) for COVAM and ELISA, respectively. (C and D) Patients with αEp9 Abs are symptomatic for longer durations (C) and spend more days in the hospital (D) than those with other αN Abs or no αN Abs. ANOVA with Tukey’s multiple comparisons yields *P* values of <0.05 (*) and <0.01 (**). One outlier (black) (robust regression and outlier removal = 0.1%) was omitted from statistical calculations for panels C and D. (E) The αN IgG appears at high levels early in the course of disease only for αEp9-positive patients and is lower in non-Ep9, αN-positive patients. After >15 days post-symptom onset, αN IgG levels increase for both groups of patients. (F) However, IgM levels do not change significantly. Error bars depict SEM with the indicated number of patients (*n*, numbers above columns).

Direct comparison of data with full-length N protein from COVAM and Ep9 phage ELISA (*n* = 40 patients assayed with both techniques) revealed five unique categories of patients (Fig. 3A). To enable this comparison, raw data from each assay were normalized as a percentage of the negative control. Category 1 consists of patients without Abs to the N protein. The next categories included patients with IgMs (category 2) or IgGs (category 3) binding to N protein, but not Ep9, termed non-Ep9 αN Abs. Category 4 included patients with αEp9 Abs (both IgMs and IgGs). Category 5 patients had exclusively IgG αEp9 Abs. The αEp9 Abs were found only in patients with IgMs or IgGs against full-length N protein from the COVAM assay; the COVAM analysis thus independently corroborated the phage ELISAs (Fig. 3A).

Interestingly, the patients with αEp9 Abs suffered more prolonged illness and worse clinical outcomes compared to patients with non-Ep9 αN Abs or no αN Abs. In this study, severe COVID-19 cases were defined as resulting in death or requiring admission to the intensive care unit (ICU) or intubation. The fraction of severe COVID-19 cases was 2.5 times higher in αEp9 Abs patients than non-Ep9 αN Abs patients (Fig. 3B, yellow panel); the differences in proportions of severe and nonsevere αN-positive patients with or without αEp9 Abs were statistically significant (*P* < 0.030, Fisher’s exact test). Patients without αN Abs (category 1) had less severe symptoms. The αEp9 Ab patients also had longer durations of symptoms and hospital stays relative to patients with non-Ep9 αN Abs or no αN Abs (Fig. 3C and D). A larger data set of patient plasma analyzed by phage ELISA confirmed this conclusion (*P* < 0.0013, Fisher’s exact test) (Fig. 3B, blue panel). Our data further demonstrated that asymptomatic COVID-19 patients (*n* = 3) also tested negative for αEp9 Abs (Table S2). The data also revealed early seroconversion of αEp9 IgGs (Fig. 3E) but not αEp9 IgMs (Fig. 3F).

10.1128/mSphere.00203-21.9 TABLE S2 Demographics and clinical characteristics of COVID-19 patients categorized by αEp9 Ab response. Download Table S2, PDF file, 0.2 MB. Copyright © 2021 Sen et al. 2021 Sen et al. https://creativecommons.org/licenses/by/4.0/ This content is distributed under the terms of the Creative Commons Attribution 4.0 International license.

### Strong correlation between disease severity and comorbidities in patients with αEp9 Abs.

**FIG 4:**
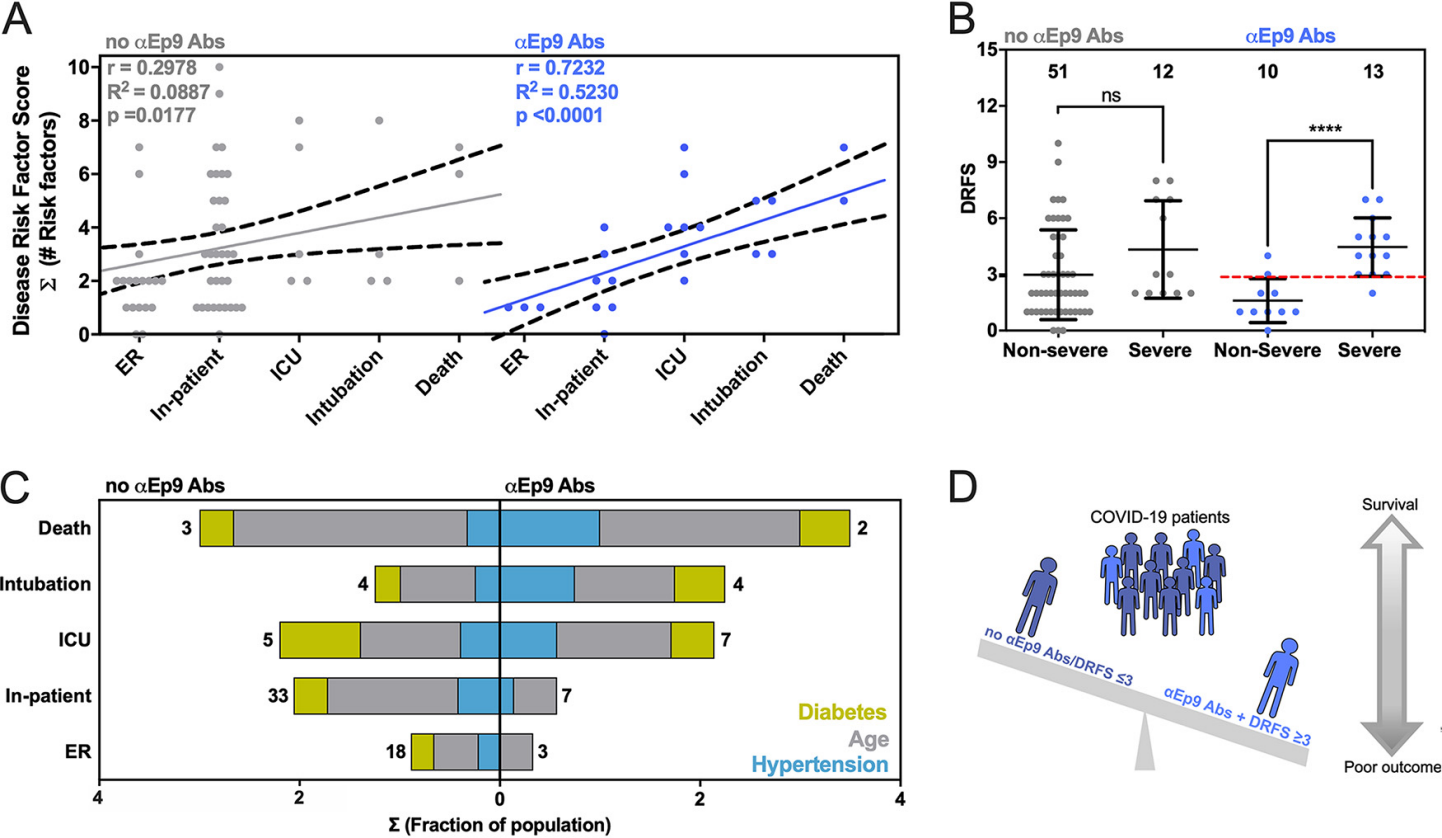
Correlation between disease severity and risk factors in patients with αEp9 Abs. (A) The relationship between DRFS and disease severity of COVID-19 patients with αEp9 Abs (blue) or no αEp9 Abs (gray). Each data point represents one patient. The solid lines indicate linear regression fits with 95% confidence intervals (dashed lines) and Pearson’s *r* value as noted. (B) Correlation of disease severity with DRFS in patients with αEp9 Abs. The data depict a significant correlation between DRFS and disease severity in patients with αEp9 Abs (blue) but not in patients lacking αEp Abs (gray). In αEp9 patients, a DRFS threshold of 3.0 can predict severe disease (red). Two-tailed, parametric *t* tests were conducted to compare nonsevere and severe disease outcomes of patients with and without αEp9 Abs, where **** indicates *P* < 0.0001. The error bars represent SD with the indicated *n*. (C) The color-indicated risk factors (diabetes, hypertension, and age score) are depicted on the *x* axis as the fractions of patients in each disease severity category (*y* axis). Numbers indicate total patients (*n*) without αEp9 Abs (left) or with αEp9 Abs (right). The prevalence of risk factors (colors) increases with disease severity in patients with αEp9 Abs but not in patients without these Abs. (D) Patients with αEp9 Abs and DRFS of ≥3 are predisposed to increased COVID-19 severity and poorer outcomes.

We compared risk factors, clinical parameters, and disease outcomes among patients with αEp9 Abs (*n* = 23) (Fig. 4A and Fig. S5). A disease risk factor score (DRFS) was developed to evaluate the relationship between clinical preconditions and disease severity in patients with αEp9 Abs. The DRFS quantified a patient’s age, sex, and preexisting health conditions associated with COVID-19 disease severity and mortality. Risk factors include hypertension, diabetes, obesity, cancer, and chronic conditions of the following kinds: cardiac, cerebrovascular, and kidney (26–29). Using the age score from the Charlson comorbidity index (30) yields a patient’s DRFS as DRFS = Σ (number of risk factors) + (age score), where each risk factor was valued as either 0 or 1 if absent or present, respectively. The DRFS of patients with αEp9 Abs strongly correlated with COVID-19 disease severity (Pearson’s *r* = 0.72, *P* value < 0.0001, and *R*^2^ = 0.52) (Fig. 4A). The correlation in patients without αEp9 Abs was weak (*r* = 0.30, *P* value = 0.089, *R*^2^ = 0.018) (Fig. 4A). Among patients with αEp9 Abs (*n* = 23), a DRFS of ≥3 determined disease severity with 92.3% sensitivity (1/13 false negatives) and 80% specificity (2/10 false positives) (Fig. 4B). In the entire study cohort (*n* = 86), patients with αEp9 Abs and a DRFS of ≥3 (*n* = 11) have severe disease with a high degree of specificity (96.7%) and a sensitivity of 44%. Notably, DRFS predicted disease severity only for patients with αEp9 Abs (*n* = 23), and patients without such Abs (*n* = 63) had no correlation with disease outcomes.

10.1128/mSphere.00203-21.5 FIG S5 Comparison of disease severity and clinical parameters of patients with αEp9 Abs. The data shown represent the fold change of each clinical parameter over the mean of the normal range. The sum of all the fold changes of the clinical parameters for each Ep9-responsive patient is binned according to COVID-19 disease severity. For facile visualization and comparison of clinical biomarkers between Ep9-reponsive patients, the values of each parameter were normalized to fold over the mean of healthy values. No significant trends in clinical parameters (color indicated) were observed with increased disease severity or relative to patients lacking αEp9 Abs. Download FIG S5, PDF file, 0.6 MB. Copyright © 2021 Sen et al. 2021 Sen et al. https://creativecommons.org/licenses/by/4.0/ This content is distributed under the terms of the Creative Commons Attribution 4.0 International license.

Examining key contributors to high DRFS, the presence of αEp9 Abs correlated with more severe disease in patients who have hypertension, diabetes, or age of >50 years. Such correlation was not observed for patients lacking αEp9 Abs (Fig. 4C). Such risk factors were prevalent at roughly the same percentages in the two populations of patients (Table S2). Thus, these risk factors were particularly acute for patients with αEp9 Abs.

### High levels of inflammatory cytokine and tissue damage markers in patients with αEp9 Abs.

**FIG 5:**
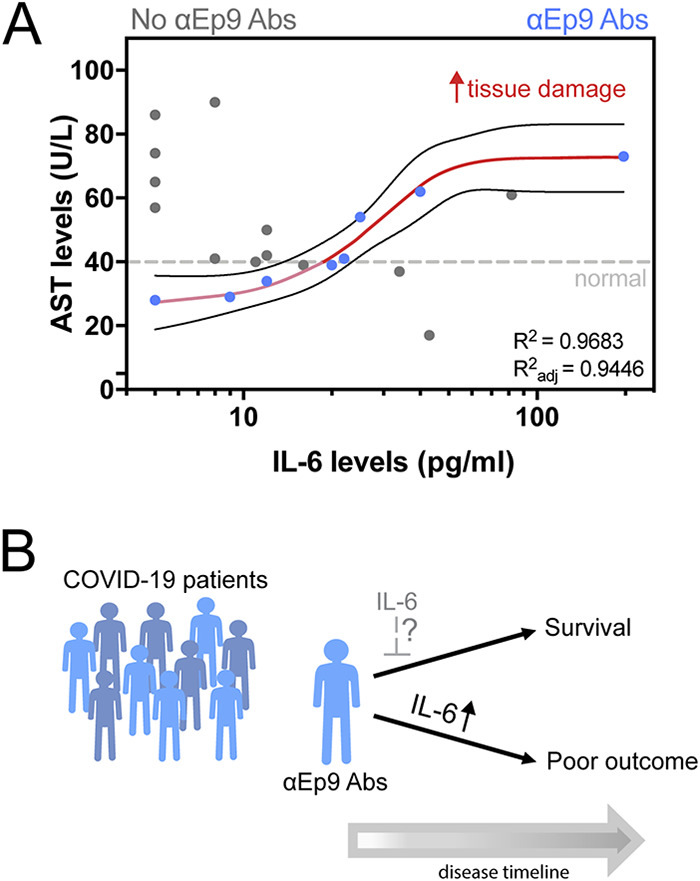
Association of inflammatory cytokine and tissue damage markers in patients with αEp9 Abs. (A) Association between the inflammatory cytokine IL-6 and the tissue damage marker aspartate transaminase (AST) shows a sigmoidal curve fit for patients with αEp9 Abs, *R*^2^ = 0.9683, Spearman’s correlation coefficient = 1.0, *P* < 0.0001. (B) Schematic of patients with αEp9 Abs with increasing IL-6 levels leading to poor outcomes. We hypothesize that patients with αEp9 Abs could benefit from IL-6 inhibition early in the disease, such as monoclonal antibody drugs targeting IL-6 or its receptor (IL6R), to disrupt a cytokine storm and reduce severe outcomes.

COVID-19 patients can have elevated serum concentrations of >20 inflammatory cytokines and chemokines (31). However, information on the cytokine levels and the association with tissue damage and worse COVID-19 outcomes has been inconsistent (31–33). For patients with IL-6 concentrations measured in plasma, patients with (*n* = 8) or without (*n* = 11) αEp9 Abs were compared. Interestingly, the comparison uncovered a strong positive sigmoidal association between IL-6 and AST unique to patients with αEp9 Abs (*R*^2^ = 0.968, Spearman’s *r* = 1.0, *P* value < 0.0001, *n* = 8) (red line, Fig. 5A); correlation of IL-6 and AST in patients with αEp9 Abs remained strong even after removal of the data point at the highest IL-6 concentration. Conversely, a slight negative trend was observed in patients lacking αEp9 Abs (Spearman’s *r* = −0.575, *P* value = 0.0612, *n* = 13). Thus, the presence of αEp9 Abs could disambiguate the sometimes-contradictory association of IL-6 with disease severity.

## DISCUSSION

This study introduces a two-step test as a prognostic for predicting COVID-19 disease severity and its worst outcomes. Specifically, αEp9 Abs can effectively predict severe disease (specificity, 83.6%). However, combining presence of αEp9 Abs with DRFS of ≥3 provides much higher specificity (96.7%) for predicting severe disease. Previously, αN IgGs have been recognized as a focal site for an antibody response (18, 19, 21, 34) and associated with disease severity and poor outcomes (11, 34, 35).

The present investigation expands on previous reports that recognize various regions of the RNA binding domain of N protein as focal sites for anti-SARS-CoV-2 antibody response. For example, the phage display-based VirScan identified an epitope region spanning residues 141 to 196 and microarrays further isolated peptides including residues 134 to 171, 155 to 171, 153 to 190, and 153 to 171 (18, 19, 21). The above investigations, however, do not find correlations between any of these epitopes and disease severity. Our results are confirmed by observations from a patient cohort in Singapore, which identify an epitope (residues 153 to 170) very similar to Ep9 (residues 152 to 172) and show a correlation between antibody response against the epitope and pneumonia and the tissue damage markers (CRP and lactate dehydrogenase [LDH]) (20). In our investigation, we examine in-depth patient clinical histories, test results, disease outcomes ranging from asymptomatic to fatal, and longer longitudinal profiling post-symptom onset, to determine the association of a larger subset of markers and risk factors. Such data allow calculation of the DRFS. Together with the presence of αEp9 Abs, patient DRFS allows early discrimination of severe from nonsevere disease outcomes. Additionally, fine epitope mapping demonstrates that αEp9 Abs strongly and uniquely correlate with COVID-19 disease severity relative to other αN Abs.

We hypothesize that the underlying mechanism relating αEp9 Abs to increased disease severity involves an overzealous immune response. Specifically, we observe early seroconversion and strong early upregulation of αEp9 IgGs (Fig. 3E). Similar IgG observations have been correlated with poor viral neutralization and clearance, resulting in increased COVID-19 severity (10, 35, 36). Also, high levels of IL-6 are observed for αEp9-positive patients with increased levels of the tissue damage marker AST; this correlation does not exist for patients lacking αEp9 Abs (Fig. 5A). The sensitivity to IL-6 concentration before AST-monitored organ damage suggests anti-IL-6 therapeutics could be an effective tool for management in the early and rapidly progressive stages of respiratory distress for αEp9-positive patients (31, 37–41). Since binding to N protein by αEp9 antibodies is unlikely to enhance uptake of SARS-CoV-2, an antibody-dependent enhancement mechanism could invoke antigen uptake by macrophages. This mechanism could stimulate complement activation and the cytokine storm observed here as elevated IL-6 response. Further investigation is required to determine the basis for increased disease severity in αEp9 patients.

The data demonstrate that αEp9-positive patients with a DRFS of ≥3 are 13.42 times (likelihood ratio) more likely to have severe COVID-19 disease symptoms within the study cohort (*n* = 86). The presence of αEp9 without DRFS is less effective as a prognostic (likelihood ratio of 3.17). Despite its high specificity (96.7%), the sensitivity of this two-step test is 44% (*n* = 86). However, this test could predict a subset of patients with a specific immune response (i.e., early IgG response and IL-6-dependent immune hyperactivity), and could suggest targeted treatment options (e.g., targeting IL-6 and its pathways).

Importantly, αEp9 Abs appear early in the course of disease. Thus, such a prognostic could outperform traditional markers for the cytokine storm such as IL-6, which appears 6 to 8 days after symptom onset (31, 39); all plasma samples collected from αEp9-positive patients (*n* = 7, Fig. 2D) between 1 and 6 days post-symptom onset demonstrate detectable levels of αEp9 IgG (≥2-fold over negative control). Early detection of αEp9 Abs in patients could be used to triage and treat COVID-19 prior to the onset of its most severe symptoms; delayed treatments with IL-6-targeting drugs can decrease their efficacy or be counterproductive (31, 37–42) (Fig. 5B). The αEp9 Ab biomarker could identify patients most likely to benefit from anti-IL-6 therapeutics and avoid ineffective treatments.

This study demonstrates the usefulness of fine epitope mapping, but the following limitations should be noted. Short linear epitopes, unlike conformational epitopes in larger domains, might not resemble the tertiary structure of an antigen. Posttranslational modifications, such as glycosylation, were omitted for the phage-displayed S protein epitopes; the COVAM antigens, however, are produced in baculovirus or HEK-293 cells, which could glycosylate the antigens. Our analysis is largely based upon a population of 86 COVID-19 patients and 5 healthy individuals, with the majority being of Hispanic descent. The conclusions could be further strengthened with follow-up investigations in a larger population. Additionally, the population examined here included only three asymptomatic individuals, and additional testing is required to verify the absence of αEp9 Abs in such patients. The sample size of patients with multiple antibody targets was too limited to allow correlation analysis; future investigations could examine associations between αEp9 and other Abs. Abs recognizing other SARS-CoV-2 structural proteins could also exhibit characteristics similar to αEp9 Abs.

Existing diagnostic platforms could readily be adapted to test for αEp9 Abs (e.g., assay with eGFP-Ep9 fusion demonstrated here), and the DRFS calculation is quite simple to implement. As shown here, αEp9 Abs do not recognize orthologous sequences from closely related coronaviruses, providing good specificity for αEp9 as a prognostic. Previous studies have shown that the high homology of N protein among related coronaviruses can lead to high false-positive rates in serodiagnostics with full-length N antigen (43). Thus, the two-step prognostic reported here could mitigate the worst outcomes of COVID-19, particularly for patients at high risk.

## MATERIALS AND METHODS

### Cloning.

For phage display of epitopes, the pm1165a phagemid vector as previously described (44) was engineered to encode an N-terminal FLAG tag and a C-terminal fusion to the P8 coat protein of M13 phage. This template, termed FlagTemplate, was used for subcloning of SARS-CoV-2, SARS, MERS, HKU-1, and NL63 epitopes. A vector map of the FlagTemplate (see Fig. S6A in the supplemental material), cloning procedures, and a list of oligonucleotides (Table S3) for Q5 site-directed mutagenesis and Gibson assembly are provided.

10.1128/mSphere.00203-21.6 FIG S6 Schematic of plasmid map for phage-displayed or recombinant expression epitopes. (A) Schematic of the FlagTemplate phagemid used for cloning phage-displayed epitopes. The phagemid, termed FlagTemplate, for the subcloning of SARS-CoV-2, SARS, HKU-1, and NL63 epitopes encodes an N-terminal FLAG tag, followed by a GSG linker to the epitope before a C-terminal GGGSGSSS linker to the P8 coat protein of M13 phage. (B) The plasmid map for recombinant expression of the eGFP-Ep9 fusion. Ep9 was subcloned into a pET28-eGFP fusion vector with a C-terminal FLAG tag. The fusion protein is connected through a linker (GGGSGSS), and two spacers flank the N and C termini of the FLAG tag (SGSG and GSG, respectively). The plasmid backbone lacking the Ep9 sequence was used to express the eGFP-FLAG negative control. Download FIG S6, PDF file, 0.1 MB. Copyright © 2021 Sen et al. 2021 Sen et al. https://creativecommons.org/licenses/by/4.0/ This content is distributed under the terms of the Creative Commons Attribution 4.0 International license.

10.1128/mSphere.00203-21.10 TABLE S3 Oligonucleotides used for cloning of phage-displayed putative epitope for SARS-CoV-2 and ortholog Ep9 epitope from SARS, MERS, HKU-1, and NL63. Download Table S3, PDF file, 0.3 MB. Copyright © 2021 Sen et al. 2021 Sen et al. https://creativecommons.org/licenses/by/4.0/ This content is distributed under the terms of the Creative Commons Attribution 4.0 International license.

Short (approximately 30 amino acids) putative epitopes for phage display and Escherichia coli expression as eGFP fusion peptides in the pET28 vector were cloned via Q5 site-directed mutagenesis according to the manufacturer’s instructions. A vector map of the peptide with Ep9 fused to eGFP, termed eGFP-Ep9, is shown in Fig. S6B. For large epitopes (>500 bp), such as Ep17, Gibson assembly (New England Biolabs) was conducted in two PCR steps with the FlagTemplate or pCAGGS containing the SARS-CoV-2 S protein gene (BEI Resources) to generate the vectors and inserts, respectively. The Gibson assembly (2 μl) or KLD (kinase, ligase, DpnI) mix (5 μl) was transformed into Nova Blue E. coli competent cells, and transformants were plated on a carbenicillin-supplemented (50 μg/ml) agar plate before incubation at 37°C overnight. Five single colonies were selected to inoculate 4 ml of super optimal broth (2% wt/vol tryptone, 0.5% yeast extract, 8.56 mM NaCl, 2.5 mM KCl, 10 mM MgCl_2_, 10 mM MgSO_4_) in a 15-ml culture tube supplemented with carbenicillin (50 μg/ml). The seed cultures were incubated at 37°C with shaking at 225 rpm for 8 to 12 h. Phagemid DNA was isolated using the QIAprep spin miniprep kit according to the manufacturer’s instructions. The successful subcloning of the open reading frame (ORF) encoding each epitope was verified via DNA sequencing (Genewiz). The full-length N protein in a pLVX-EF1α-IRES-Puro plasmid was a generous gift from Rachel Martin of University of California, Irvine (UCI).

### Purification and preparation of phage.

Phage were propagated and purified using procedures previously described (45) with the following changes. A single colony was selected to inoculate 15 ml of yeast extract and tryptone media (2YT) (1.6% wt/vol tryptone, 1% wt/vol yeast extract, 0.5% wt/vol NaCl) and shaken at 37°C until the optical density at 600 nm (OD_600_) reached 0.6. After incubation at 37°C for 45 min, 8 ml of the primary culture was used to inoculate 300 ml of 2YT supplemented with carbenicillin (50 μg/ml), kanamycin (20 μg/ml), and isopropyl-β-d thiogalactopyranoside (IPTG; 30 μM).

To precipitate the phage, the cultures were centrifuged at 10 krpm (15,300 × *g*) for 10 min at 4°C. The supernatant was decanted into a centrifuge tube containing 60 ml polyethylene glycol (PEG) 8000 (20%, wt/vol) and NaCl (2.5 M). The tube was inverted 10 times and stored on ice for 30 min followed by an additional centrifugation at 10 krpm (15,300 × *g*) for 20 min at 4°C. The supernatant was decanted, and tubes were centrifuged for an additional 4 min at 4 krpm (2,429 × *g*) at 4°C. The pellets were resuspended in PBS (10 mM phosphate, 137 mM NaCl, pH 7.2) with Tween 20 (0.05%, vol/vol) and glycerol (10%, vol/vol), separated into 1-ml aliquots, flash frozen with liquid nitrogen, and stored at −80°C. For binding assays via ELISA, the purified phage was thawed on ice, precipitated a second time as before. The quality of each phage preparation was routinely checked by quality control ELISA, termed QC ELISA, to a FLAG peptide fused to the N terminus of each epitope (Fig. S1); additionally, PCR using Oligo69 and Oligo70 followed by DNA sequencing (Genewiz) was performed for every phage preparation. Such quality control allowed for identification of toxic clones; for example, C8 was apparently toxic to E. coli, and three protein epitopes failed to express in E. coli for unknown reasons. The phage concentration was determined by absorbance at 260 nm using a coefficient of molar absorptivity of 0.003 nM^−1^ cm^−1^ and diluted to 40 nM in PBS.

### Expression and purification of eGFP-Ep9 and N protein.

A pET28c plasmid containing Ep9 fused to an N-terminal eGFP (Fig. S6B) was transformed into BL21(DE3)* E. coli heat shock-competent cells. A single colony was transferred to LB medium (20 ml) supplemented with kanamycin (40 μg/ml) and incubated at 37°C for 18 h. An aliquot of the starter culture (2.5 ml) was transferred to LB medium with 1% glucose (250 ml LB in a 1-liter baffled flask). After reaching an OD_600_ between 0.4 and 0.6, the culture was induced through addition of IPTG (0.5 mM) before incubation at 25°C for 18 h. The cells were centrifuged (15,300 × *g*) for 20 min at 4°C, and the cell pellet was resuspended in lysis buffer (25 mM Tris-HCl and 200 mM NaCl, pH 8.0, and supplemented with protease inhibitor cocktail) followed by sonication. The lysate was subjected to centrifugation (26,892 relative centrifugal force [rcf], 45 min, 4°C). The supernatant was incubated with charged nickel immobilized-metal affinity chromatography resin overnight on a rotary shaker (150 rpm at 4°C). The resin was equilibrated in a column and washed with wash buffer (20 mM imidazole in lysis buffer), and the purified protein was eluted using elution buffer (250 mM imidazole in lysis buffer). Elutions containing the purified protein were visualized using 10% or 12% SDS-PAGE (Bio-Rad Mini-Protean Tetra electrophoresis system) stained with Coomassie brilliant blue stain (Fig. S7). The eluted fractions containing the purified eGFP-Ep9 were pooled and buffer exchanged for 3 column volumes (20 ml) with lysis buffer without imidazole using a 10-kDa-cutoff microconcentrator (Vivaspin; Fisher Scientific). The protein concentration was determined by a bicinchoninic acid (BCA) assay or Bradford assay using the estimated molecular weight (MW) (http://www.expasy.org). Similar to eGFP-Ep9, the full-length N protein was expressed in 250 ml LB with 1% glucose and induced with 0.25 mM IPTG at an OD_600_ of 0.8. Protein overexpression cultures were incubated at 16°C for 22 h. Lysis and purification were conducted as described above, using N protein lysis buffer (20 mM Tris-HCl, 300 mM NaCl, 5 mM MgCl_2_, 5 mM β-mercaptoethanol [BME], 10% glycerol, pH 8.0). The purified full-length N protein was analyzed using 10% SDS-PAGE (Fig. S7).

10.1128/mSphere.00203-21.7 FIG S7 eGFP-FLAG, eGFP-Ep9, and full-length N protein purity assessed by 10% SDS-PAGE. Representative SDS-PAGE to visualize eGFP-FLAG and eGFP-Ep9 (A) or full-length N protein (B) after immobilized-metal (Ni^2+^) affinity chromatography purification. Elution fraction 2 of eGFP-FLAG, eGPF-Ep9, and elution fraction 3 were purified to >95% homogeneity and migrated at the expected masses of ≈32, ≈34, and ≈48 kDa, respectively. Elution fraction 1 of eGFP-FLAG and eGFP-Ep9 was diluted 20-fold. L1, BLUE stain (Goldbio), and L2, Prestain PAGE-Ruler Plus (Thermo Fisher Scientific) protein ladder, were used as reference. I, insoluble fraction; S, soluble fractions; FT, flowthrough; W, wash. Download FIG S7, PDF file, 0.05 MB. Copyright © 2021 Sen et al. 2021 Sen et al. https://creativecommons.org/licenses/by/4.0/ This content is distributed under the terms of the Creative Commons Attribution 4.0 International license.

### Patient sample collection.

The UCI Experimental Tissue Resource (ETR) operates under a blanket IRB protocol (UCI no. 2012-8716) that gives ETR personnel “Honest Broker” status and enables the collection of any fluid or tissue remnant in excess of that needed for clinical diagnosis and distribution to investigators under the conditions of their own IRB approval. Patients undergoing COVID testing in the Emergency Department or on the inpatient service with confirmed COVID^+^ pharyngeal swabs were followed for their blood collections daily. Specimens collected originally for diagnostic purposes were processed and stored by the hospital laboratory in a manner compliant with College of American Pathologists (CAP) standards. EDTA-anticoagulated whole blood was stored for 2 days at 4°C after clinical diagnosis and released for research purposes. Plasma from heparin-anticoagulated blood was centrifuged immediately after collection and preserved at 4°C for 3 to 4 days before being released for research use. All COVID^+^ specimens were handled under biosafety level 2 (BSL-2) conditions, aliquoted into screw-cap cryovials, and stored at −80°C long term with constant temperature monitoring. Specimens were coded by the ETR with unique deidentifiers, and accompanying clinical information was stripped of protected health information such that investigators could receive specimens under a Non-Human Subjects Determination exemption from the UCI IRB. All samples from SARS-CoV-2-infected patients were inactivated by incubation in a water bath at 56°C for 30 min (46), aliquoted (40 μl each), and stored at −80°C.

### Phage ELISA with plasma.

The phage-displayed SARS-CoV-2 epitopes were used in phage ELISAs with patient plasma samples diluted 100-fold in coating buffer (50 mM Na_2_CO_3_, pH 9.6). After incubation in a 96-well Nunc MaxiSorp flat-bottom microtiter plate with shaking at 150 rpm at 4°C for 12 to 18 h, plasma was aspirated by a plate washer (BioTek). Next, the plate was treated with 100 μl per well of ChonBlock blocking/sample dilution buffer (Chondrex, Inc.) for 1 h with shaking at 150 rpm at room temperature and washed three times with wash buffer (0.05% [vol/vol] Tween 20 in PBS). The epitope displaying phage and controls were diluted to 1 nM in ChonBlock blocking/sample dilution buffer, and 100 μl was added to each well before incubating for 2 h with shaking (150 rpm) at room temperature. The plate was then washed three times with wash buffer. The primary antibody, anti-M13-horseradish peroxidase (HRP) (Creative Diagnostics), was diluted 1:5,000 in ChonBlock secondary antibody buffer, and 100 μl was added per well; the plate was incubated for 1 h at 150 rpm and room temperature. Following three washes with wash buffer, 1-Step Ultra 3,3′,5,5′-tetramethylbenzidine (TMB)-ELISA substrate solution (100 μl per well; Thermo Scientific) was added. Absorbance of TMB substrate was measured twice at 652 nm by a UV-visible (UV-Vis) plate reader (BioTek) after 5 and 15 min of incubation.

### ELISA of eGFP-Ep9 and full-length N protein with plasma.

Various doses, with a maximum concentration of 1.7 μM, of eGFP-Ep9, eGFP-FLAG, or full-length N protein (fl-N) were diluted in PBS (pH 8.0) and then immobilized on a 96-well Nunc MaxiSorp flat-bottom microtiter plate before incubation on a shaker (150 rpm) at 4°C for 12 to 18 h. After incubation, unattached proteins were removed through aspiration using a plate washer (BioTek) and wells were blocked with 100 μl ChonBlock blocking/sample dilution buffer (Chondrex, Inc.) for 30 min with shaking (150 rpm) at room temperature. The plate was then washed three times with wash buffer (0.05% [vol/vol] Tween 20 in PBS). Pooled plasma from five patients within each experimental group was diluted 100-fold in ChonBlock blocking/sample dilution buffer, and 100 μl was added to each well before incubating for 1 h with shaking (150 rpm) at room temperature. The plate was then washed three times with wash buffer. The detection antibody, IgG Fc goat anti-human–HRP (Invitrogen), was diluted 1:5000 in ChonBlock secondary antibody buffer, and 100 μl was added per well; the plate was incubated for 30 min at 150 rpm and room temperature. Following six washes with wash buffer, 1-Step Ultra TMB-ELISA substrate solution (100 μl per well; Thermo Scientific) was added. Absorbance of TMB substrate was measured twice at 652 nm by UV-Vis plate reader (BioTek) after 5 and 15 min of incubation.

### COVAM.

Serum coronavirus antigen microarray (COVAM) included 67 antigens across respiratory virus subtypes including 11 antigens from SARS-CoV-2 expressed in either baculovirus or HEK-293 cells as previously detailed (22). These antigens were provided by Sino Biological U.S. Inc. as either catalog products or custom synthesis service products. The antigens were printed onto microarrays, probed with human sera, and analyzed as previously described (47–49). Briefly, lyophilized antigens were reconstituted with sterile water to a concentration of 0.1 mg/ml protein in PBS, and printing buffer was added. Antigens were then printed onto Oncyte Avid nitrocellulose-coated slides (Grace Bio-Labs) using an OmniGrid 100 microarray printer (GeneMachines). The microarray slides were probed with human sera diluted 1:100 in 1× protein array blocking buffer (GVS Life Sciences, Sanford, ME) overnight at 4°C and washed with TTBS buffer (20 mM Tris-HCl, 150 mM NaCl, 0.05% Tween 20 in double-distilled water (ddH_2_O) adjusted to pH 7.5 and filtered) three times for 5 min each. A mixture of human IgG and IgM secondary antibodies conjugated to quantum dot fluorophores Q800 and Q585, respectively, was applied to each of the microarray pads and incubated for 2 h at room temperature, and pads were then washed with TTBS three times for 5 min each and dried. The slides were imaged using an ArrayCam imager (Grace Bio-Labs) to measure background-subtracted median spot fluorescence. Nonspecific binding of secondary antibodies was subtracted using a saline control. The mean fluorescence of the 4 replicate spots for each antigen was used for analysis.

### Statistical analysis.

The ELISA data were analyzed in GraphPad Prism 8. Since the total antibody content differs from person to person, the raw absorbance values for every patient sample were normalized and represented as the ratio compared to a negative control. Analysis of variance (ANOVA) with Dunnett’s multiple-comparison test was performed to determine if values were statistically significant. Correlations between COVAM IgG/IgM and ELISA were determined by plotting normalized values on an *xy* graph and performing a nonparametric correlation analysis using a Spearman rank correlation coefficient test.

For data visualization of clinical patient data, trends in data were evaluated using Knime Analytics Platform software. GraphPad Prism was used to calculate column statistics including mean, standard deviation, standard error of the mean (SEM), *P* values, odds ratios, and likelihood ratios defined as sensitivity/(1 − specificity). ANOVA with Tukey’s multiple-comparison test was used to evaluate antibody response and disease severity between patients with αEp9 Abs, non-Ep9 Abs, αN Abs, or non-αN Abs. Comparisons of patients with αEp9 Abs and non-αEp9 Abs were conducted using unpaired, two-tailed, parametric *t* tests. Contingency graphs were statistically evaluated using Fisher’s exact test, for groups with binary categorization, and the chi-squared test for groups with multiple categories. Different data sets were fitted with linear or nonlinear regression methods; the fit with the higher *R*^2^ value was chosen. Correlations between two clinical parameters (e.g., IL-6 and AST) were evaluated using the Pearson coefficient or Spearman coefficients (*r*) for linear or nonlinear regressions, respectively; *r* values between 1.0 and 0.7 were considered strong correlations, *r* values between 0.7 and 0.5 were considered moderate correlations, and values below 0.5 were considered weak correlations (50). The significance of the correlation was evaluated based on a *P* value of <0.05.

### Data availability.

Microarray data have been deposited under BioProject accession no. GSE172471.
