# Supplementary figures and images for: Predicting COVID-19 Severity with a Specific Nucleocapsid Antibody plus Disease Risk Factor Score
Source: mSphere. 2021 Apr 28;6(2):e00203-21. doi: 10.1128/mSphere.00203-21 (PMC8092137; doi:10.1128/mSphere.00203-21)

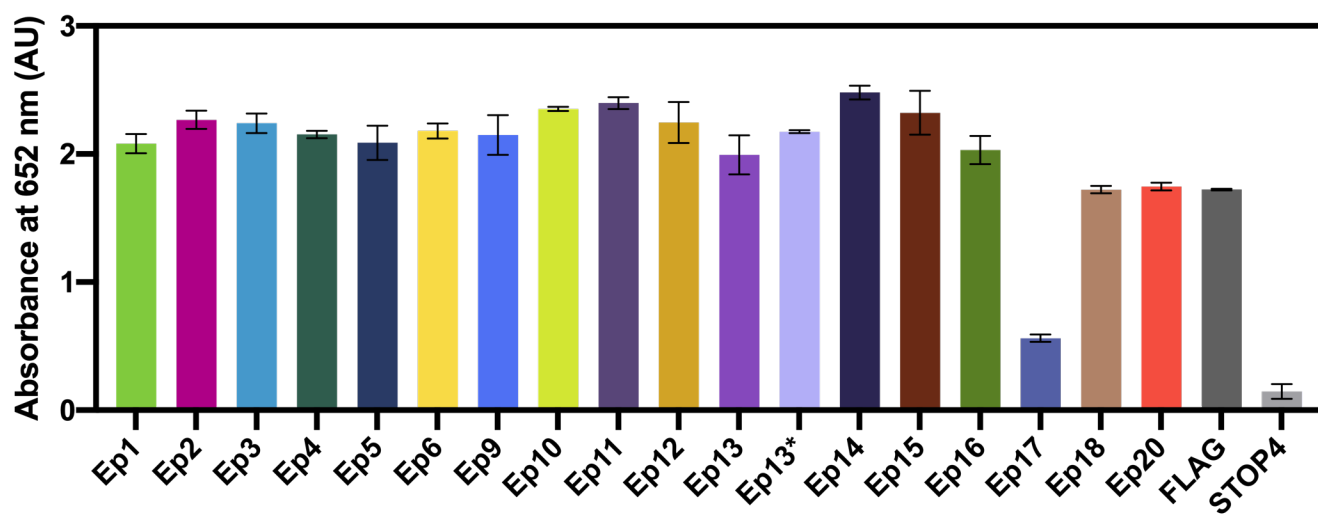

Supplement: FIG S1 [file mSphere.00203-21-sf001.pdf]

A

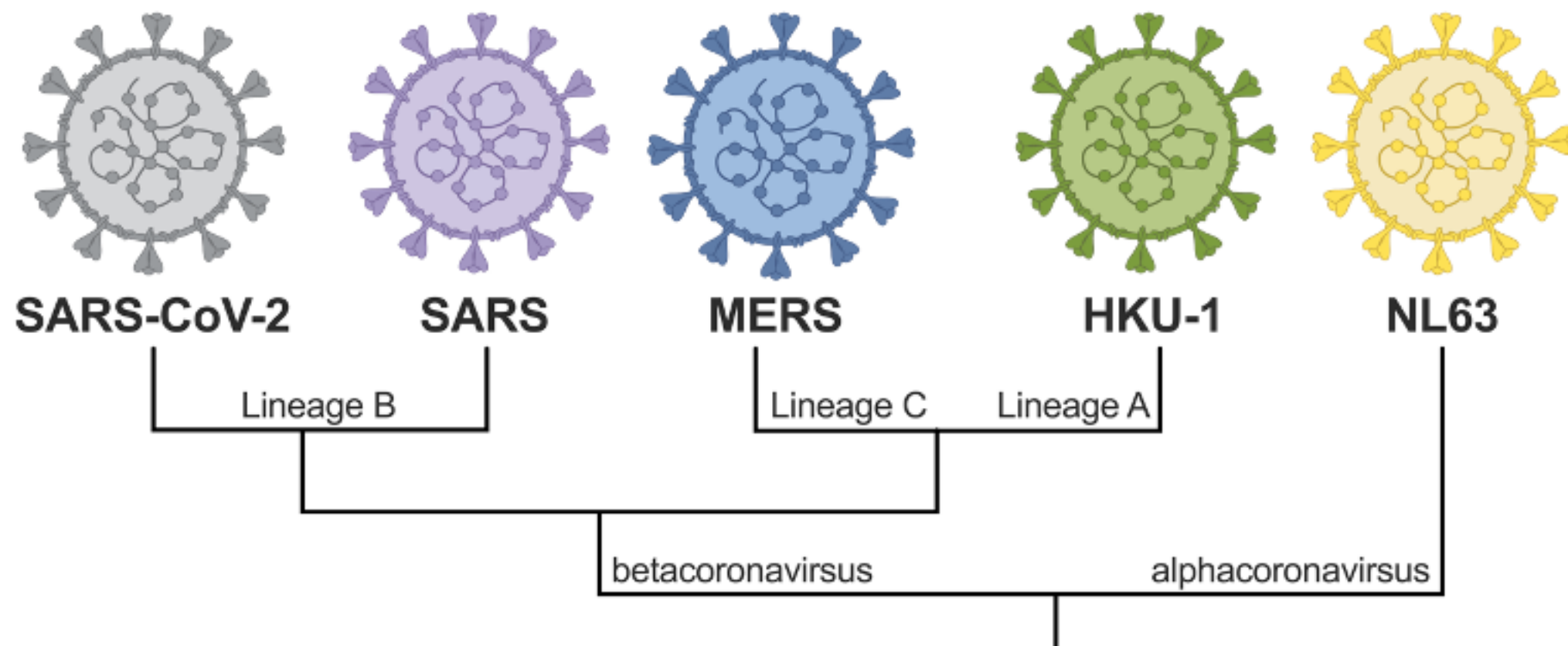

B

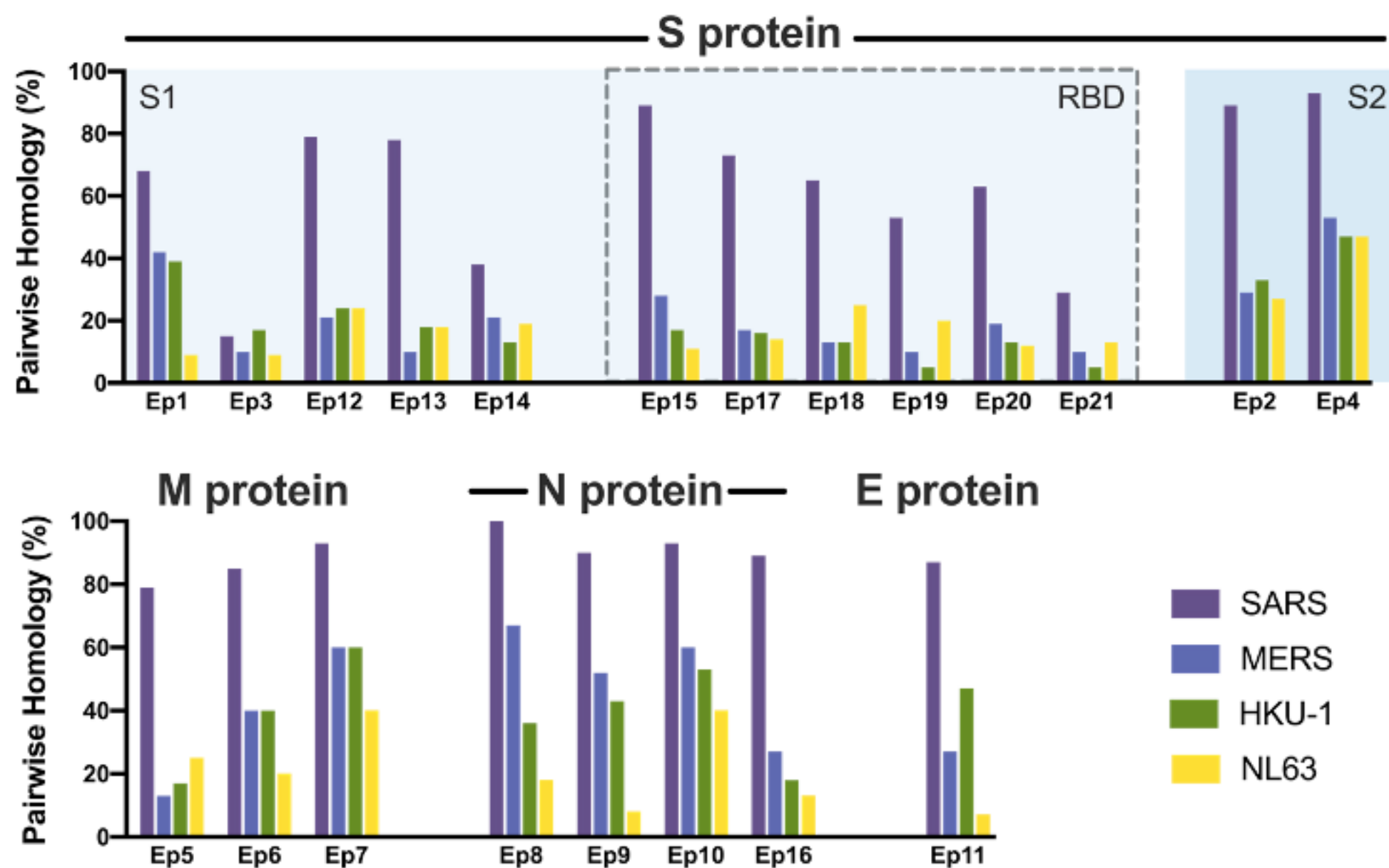

Supplement: FIG S2 [file mSphere.00203-21-sf002.pdf]

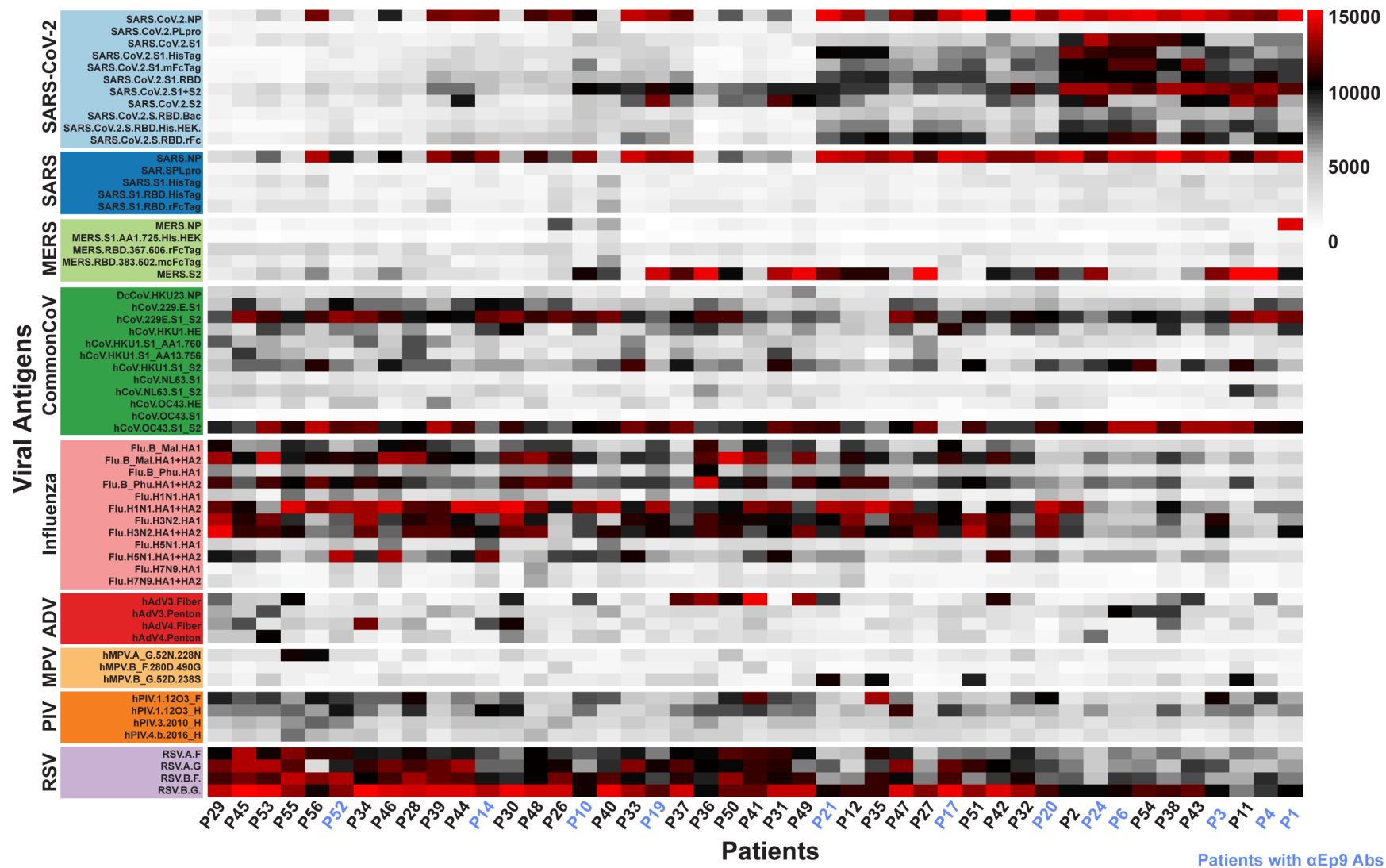

Supplement: FIG S3 [file mSphere.00203-21-sf003.pdf]

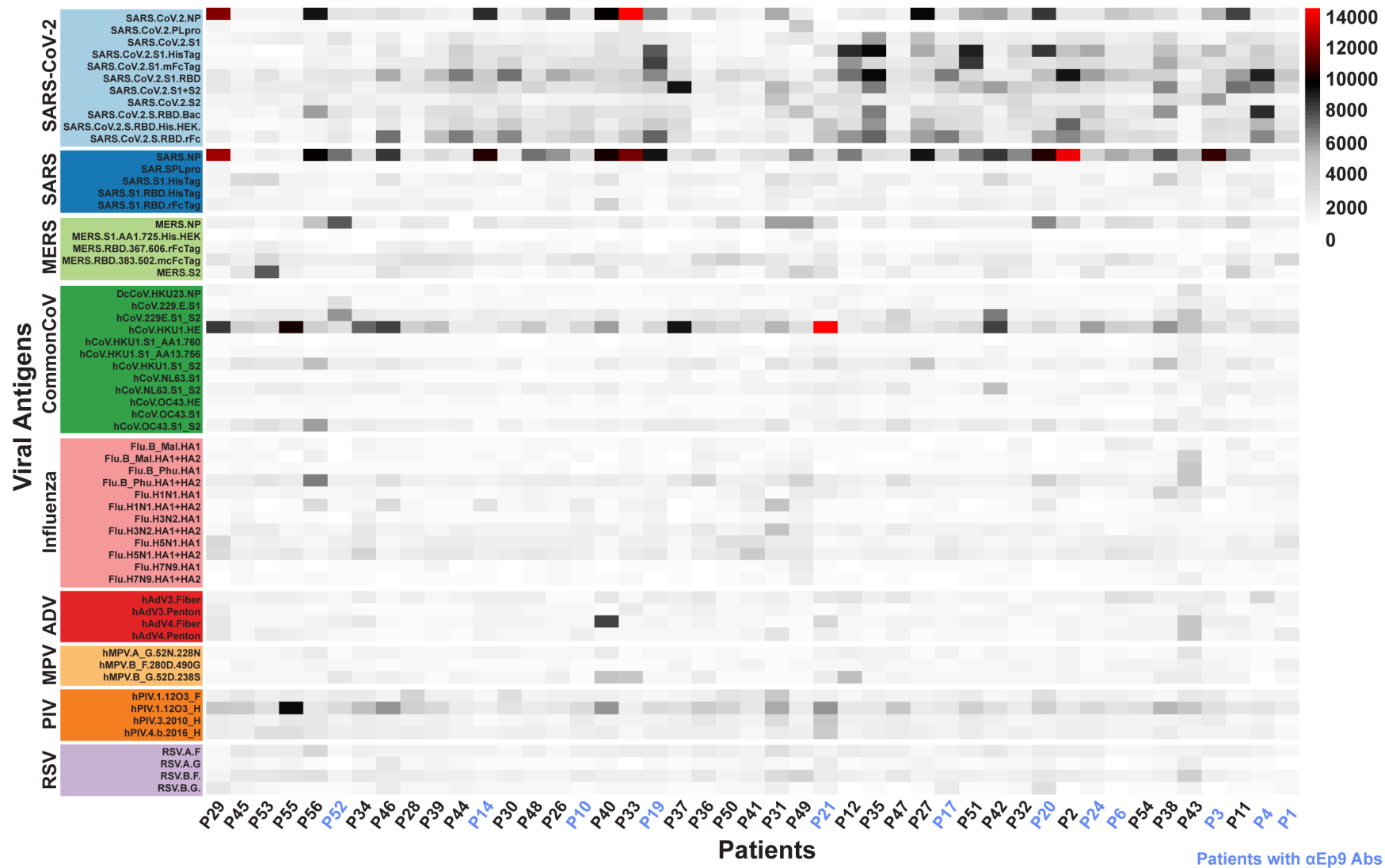

Supplement: FIG S4 [file mSphere.00203-21-sf004.pdf]

Σ Clinical Parameters (Fold over median of normal levels)

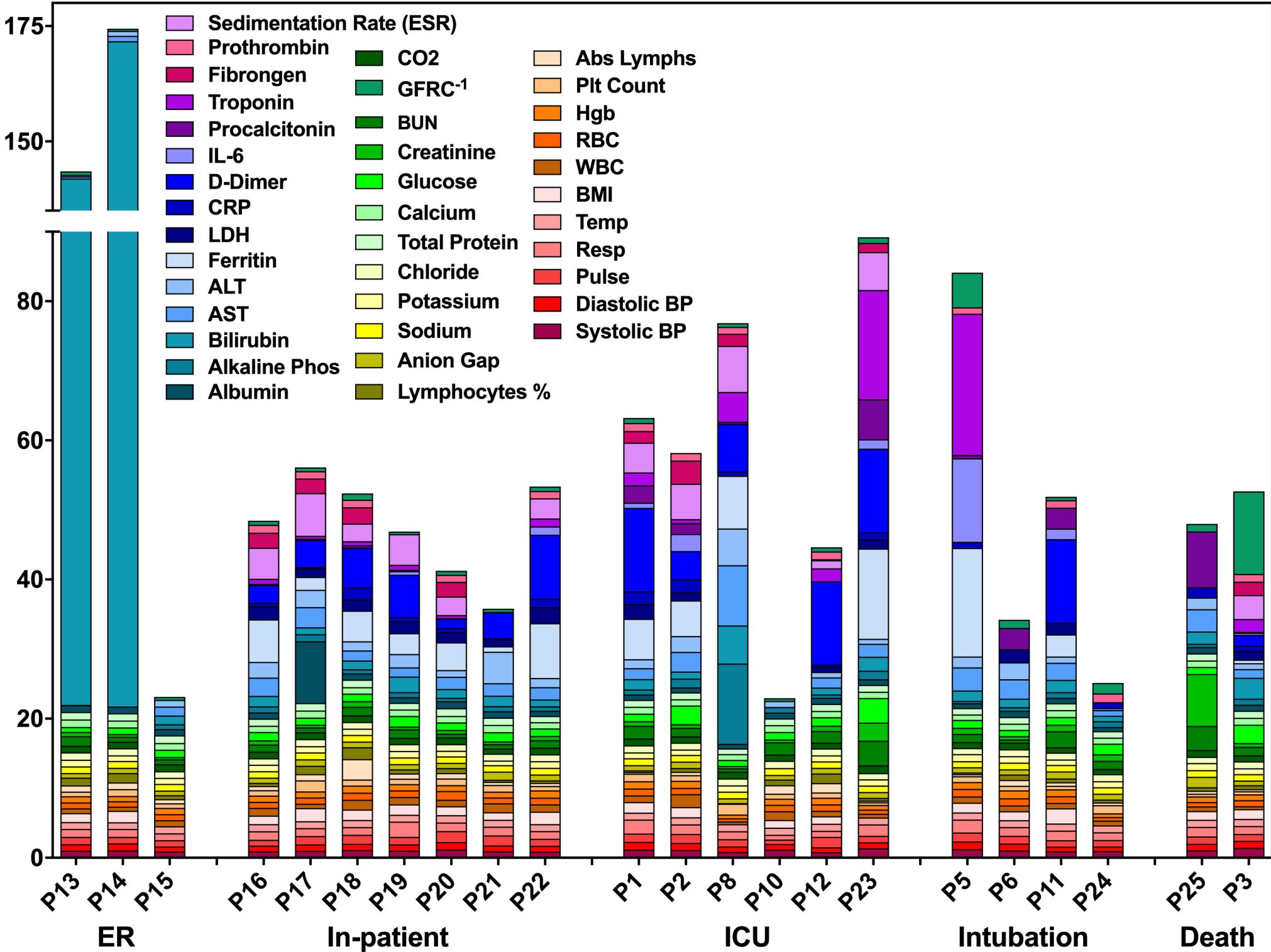

Supplement: FIG S5 [file mSphere.00203-21-sf005.pdf]

A

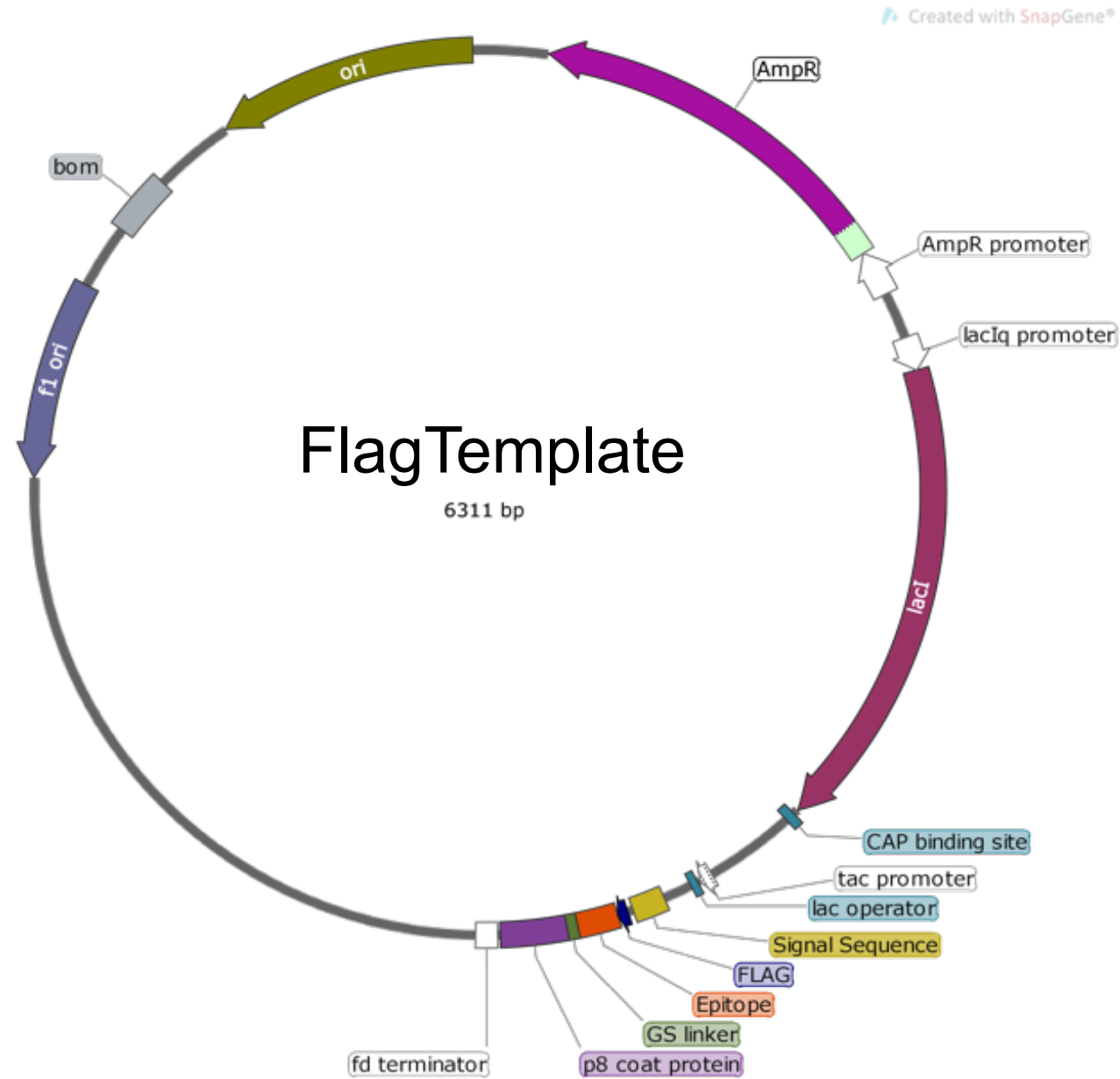

B

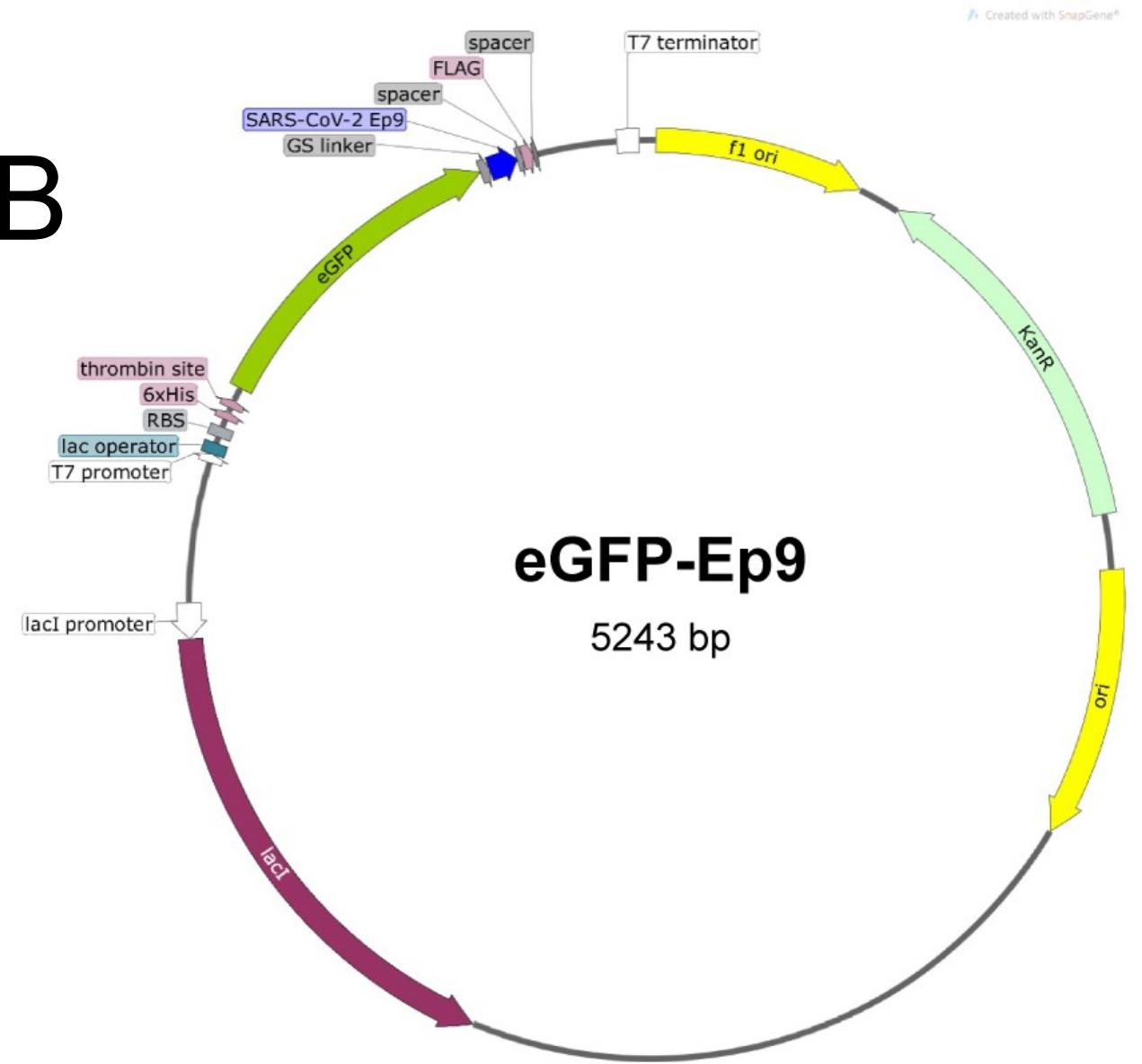

Supplement: FIG S6 [file mSphere.00203-21-sf006.pdf]

A

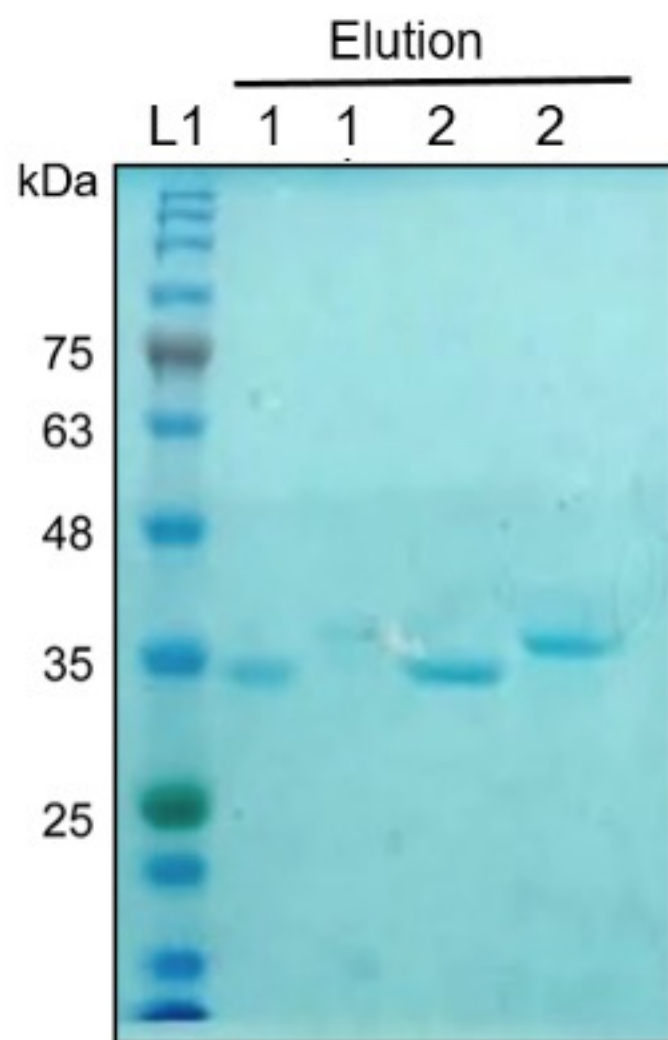

|           |   |   |   |   |
|-----------|---|---|---|---|
| eGFP-FLAG | + | - | + | - |
| eGFP-Ep9  | - | + | - | + |

B

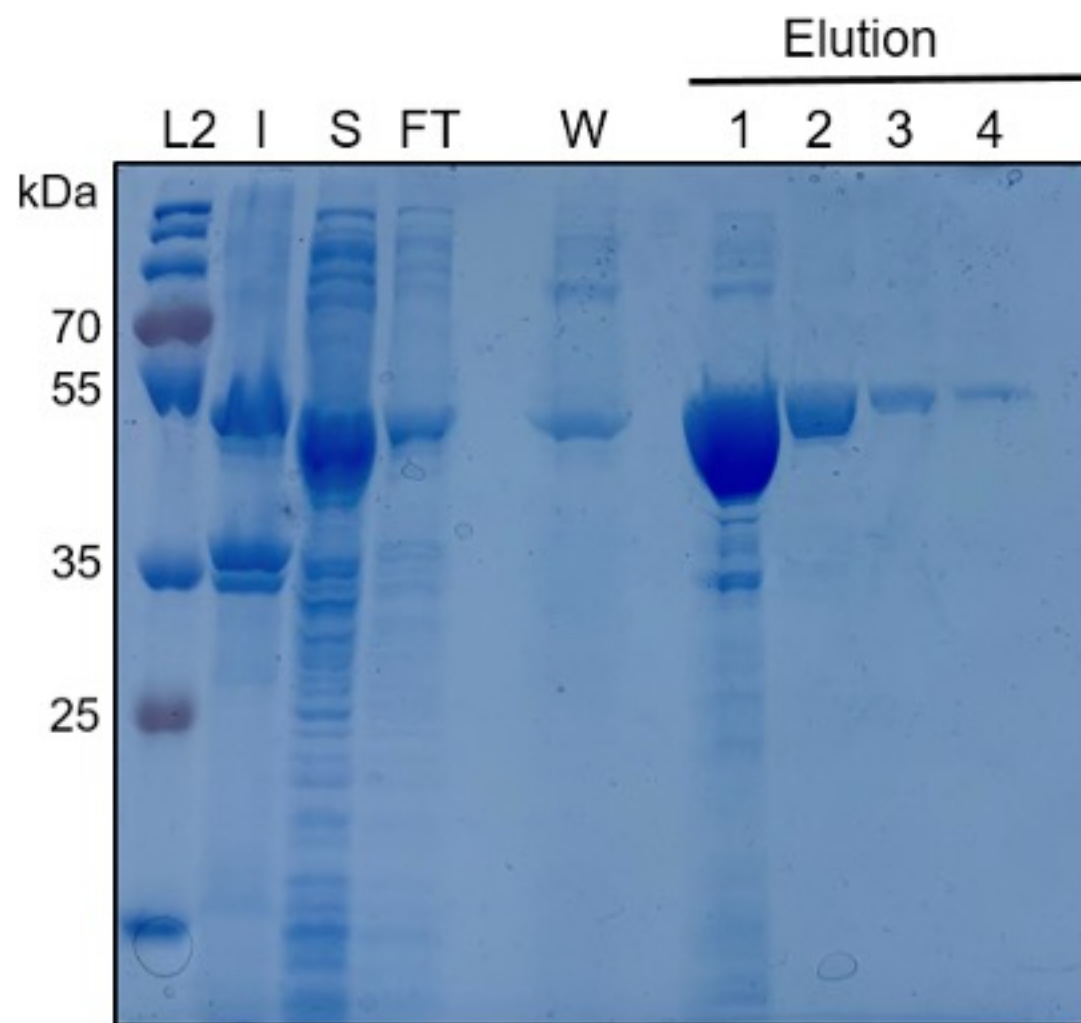

Supplement: FIG S7 [file mSphere.00203-21-sf007.pdf]
