# Supplementary material for: Predicting COVID-19 Severity with a Specific Nucleocapsid Antibody plus Disease Risk Factor Score
Source: mSphere. 2021 Apr 28;6(2):e00203-21. doi: 10.1128/mSphere.00203-21 (PMC8092137; doi:10.1128/mSphere.00203-21)
Supplement: TABLE S1 [file mSphere.00203-21-st001.pdf]

Epitope	Virus	Protein	Residues*	Amino Acid Sequence	Sequence Length	Rationale for selection	Ref. for rationale
Ep1	SARS-CoV-2	S	287-317	DAVDCALDPLSETKCTLKSFTVEKGIYQTSN	31	Predicted B-cell epitope in SARS-Cov-2 identified by bioinformatics to have immunodominant region	(*13*)
Ep2	SARS-CoV-2	S	802-819	FSQILPDPSKPSKRSFIE	18	Predicted B-cell epitope in SARS-Cov-2 identified by bioinformatics to have immunodominant region	(*13*)
Ep3	SARS-CoV-2	S	15-30	CVNLTTRTQLPPAYTN	16	Predicted B-cell epitope in SARS-CoV-2 identified by immunoinformatics	(*14*)
Ep4	SARS-CoV-2	S	1056-1070	APHGVVFLHVTYVPA	15	Predicted B-cell epitope in SARS-CoV-2 identified by structural biology and machine learning, SARS homolog of this peptide is a known B-cell epitope	(*12*)
Ep5	SARS-CoV-2	M	1-24	MADSNGTITVEELKKLLEQWNLVI	24	Predicted B-cell epitope in SARS-Cov-2 identified by bioinformatics to have immunodominant region	(*13*)
Ep6	SARS-CoV-2	M	132-151	PLLESELVIGAVILRGHLRI	20	Predicted B-cell epitope in SARS-Cov-2 identified by bioinformatics to have immunodominant region	(*13*)
Ep7	SARS-CoV-2	M	97-111	IASFRLFARTRSMWS	15	Predicted B-cell epitope in SARS-CoV-2 identified by structural biology and machine learning, SARS homolog of this peptide is a known B-cell epitope	(*12*)
Ep8	SARS-CoV-2	N	41-61	RPQGLPNNTASWFTALTQHGK	21	Predicted B-cell epitope in SARS-Cov-2 identified by bioinformatics to have immunodominant region n	(*13*)
Ep9	SARS-CoV-2	N	152-172	ANNAAIVLQLPQGTTLPKGFY	21	Predicted B-cell epitope in SARS-Cov-2 identified by bioinformatics to have immunodominant region	(*13*)
Ep10	SARS-CoV-2	N	264-278	ATKAYNVTQAFGRRG	15	Predicted B-cell epitope in SARS-CoV-2 identified by structural biology and machine learning, SARS homolog of this peptide is a known B-cell epitope	(*12*)
Ep11	SARS-CoV-2	E	52-66	VKPSFYVYSRVKNLN	15	Predicted B-cell epitope in SARS-CoV-2 identified by structural biology and machine learning, SARS homolog of this peptide is a known B-cell epitope	(*12*)
Ep12	SARS-CoV-2	S	524-598	VCGPKKSTNLVKNKCVNFNFNGLTGTGVLTESNKKFLPFQ QFGRDIADTTDAVRDPQTLEILDITPCSFGGVSVI	75	Predicted B-cell epitope in SARS-Cov-2 identified by bioinformatics to have immunodominant region	(*13*)
Ep13	SARS-CoV-2	S	601-640	GTNTSNQVAVLYQDVNCTEVPVAIHADQLTPTWRVYSTGS	40	Predicted B-cell epitope in SARS-Cov-2 identified by bioinformatics to have immunodominant region. Based on the D614G variant.	(*13*)
Ep13*	SARS-CoV-2	S	601-640	GTNTSNQVAVLYQGVNCTEVPVAIHADQLTPTWRVYSTGS	40	Sequence has the mutation D614G, which increases the fitness of SARS-CoV-2	(26,27,*54*)
Ep14	SARS-CoV-2	S	61-76	NVTWFHAIHVSGTNGT	16	Predicted B-cell epitope in SARS-CoV-2 identified by immunoinformatics	(*14*)
Ep15	SARS-CoV-2	S	373-390	SFSTFKCYGVSPTKLNDL	18	Predicted B-cell epitope in SARS-CoV-2 identified by immunoinformatics	(*14*)
Ep16	SARS-CoV-2	N	354-400	NKHIDAYKTFPPTEPKKDKKKKADETQALPQRQKKQQTVT LLPAADL	47	Predicted B-cell epitope in SARS-Cov-2 identified by bioinformatics to have immunodominant region	(*13*)
Ep17	SARS-CoV-2	S	319-529	RVQPTESIVRFPNITNLCPFGEVFNATRFASVYAWNRKRI SNCVADYSVLYNSASFSTFKCYGVSPTKLNDLCFTNVYAD SFVIRGDEVRQIAPGQTGKIADYNYKLPDDFTGCVIAWNS NNLDSKVGGNYNYLYRLFRKSNLKPFERDISTEIYQAGST PCNGVEGFNCYFPLQSYGFQPTNGVGYQPYRVVVLSFELL HAPATVCGPKK	211	Based on structure of S protein X-ray structure (PBD: 6VXX)	(*24*)
Ep18	SARS-CoV-2	S	488-507	CYFPLQSYGFQPTNGVGYQP	20	Based on X-ray structure of S protein (PBD: 6VXX)	(*24*)
Ep19	SARS-CoV-2	S	429-448	FTGCVIAWNSNNLDSKVGGN	20	Based on X-ray structure of S protein (PBD: 6VXX)	(*24*)
Ep20	SARS-CoV-2	S	448-466	NYNYLYRLFRKSNLKPFER	19	Based on X-ray structure of S protein (PBD: 6VXX)	(*24*)
Ep21	SARS-CoV-2	S	467-487	DISTEIYQAGSTPCNGVEGFN	21	Based on X-ray structure of S protein (PBD: 6VXX)	(*24*)
sEp9	SARS	N	153-173	NNNAATVLQLPQGTTLPKGFY	21	SARS homolog of Ep9	
mEp9	MERS	N	141-161	NNDSAIVTQFAPGTKLPKNFH	21	MERS homolog of Ep9	
hEp9	HKU-1	N	166-186	TTQEAIPTRFPPGTILPQGYY	21	HKU-1 homolog of Ep9	
nEp9	NL63	N	119-136	NQKPLEPKFSIALPPELS	18	NL63 homolog of Ep9	

*Residue numbering from protein sequences deposited in GenBank. Specifically, the accession numbers were as follows: S protein (YP_009724390.1), M protein (YP_009724393.1), N protein (YP_009724397.2) and E protein (YP_009724392.1) from SARS-CoV-2 and N protein from SARS (NP_828855.1), MERS (YP_009047211.1), HKU-1 (YP_173242.1), or NL63 (YP_003771.1).
