# Supplementary material for: Predicting COVID-19 Severity with a Specific Nucleocapsid Antibody plus Disease Risk Factor Score
Source: mSphere. 2021 Apr 28;6(2):e00203-21. doi: 10.1128/mSphere.00203-21 (PMC8092137; doi:10.1128/mSphere.00203-21)
Supplement: TABLE S2 [file mSphere.00203-21-st002.pdf]

Characteristics	No αEp9 Abs (n=63)	αEp9 Abs (n=23)	p-value
**Demographics**			
Age (± SD)	49.75 (± 18.45)	47.26 (± 18.45)	0.5668
Gender F: M (%)	21:42 (44.4/66.7)	10:13 (43.5/56.5)	0.4502
Ethnicity n, (%) (Hispanic: Asian: Caucasian: Black: Other)	15 (65.2): 4 (17.4): 3 (13.0): 1 (4.3): 0 (0)	39 (61.9): 8 (12.7): 9 (14.3) :3 (4.8) :4 (6.3)	0.7760
BMI (± SD)	28.89 (± 6.445)	32.06 (± 7.896)	0.0642
**Preconditions, n (%)**			
Hypertension	23 (36.5)	10 (43.5)	0.6203
Diabetes	21 (33.3)	6 (26.1)	0.6065
CVD	6 (9.5)	2 (8.7)	1.0000
CAD	6 (9.5)	2 (8.7)	1.0000
CKD/ESRD	6 (9.5)	2 (8.7)	1.0000
Asthma/COPD	8 (12.7)	3 (13.0)	1.0000
Obesity	24 (38.1)	13 (56.5)	0.1461
Cancer	2 (3.17)	3 (13.0)	0.1163
**Symptoms, n (%)**			
Total Days of Symptoms	9.8 (± 8.98)	17 (± 10.13)	0.0059**
Cough	43 (68.3)	15 (65.2)	0.7997
Dyspnea/SOB	28 (44.4)	11 (47.8)	0.8108
Myalgia/Fatigue	17 (27.0)	8 (34.8)	0.5926
Headache	12 (19.0)	2 (8.7)	0.3349
Chest pain	7 (11.1)	3 (13.0)	1.0000
Anosmia	4 (6.3)	2 (8.7)	0.6561
Stroke-like Symptoms	0	2 (8.7)	0.0692
Abdominal pain	3 (4.8)	0	0.5611
Pulmonary symptoms^ (Pneumonia: Other: None)	16 (25.4): 36 (52.38): 8 (12.7)	13 (56.5): 7 (30.4): 1 (4.3)	0.0142*
**Severity, n (%)**			
Asymptomatic	3	0	0.5611
Non-severe: Severe^^	51:12 (n, severity 19.0%)	10:13 (n, severity 56.5%)	0.0013**
Days in Hospital	5.79 (± 8.01)	10.95 (± 10.74)	0.0183*
Days in ICU	12.63 (± 13.19) n=11	12.50 (± 6.93), n=12	0.8004
Days on ventilator	14.00(± 3.96), n=6	12.86 (± 5.40), n=7	0.7934

Results are presented as mean ± standard deviation (SD) or patient number (n) and percentage of population (%). P-values for continuous variables are calculated using unpaired, two-tailed T-tests. P-values for categorical variables use Fisher's exact test for single value parameters, and Chi-squared test for multi-group variables. *, ** p-values < 0.05, 0.01, respectively.

^ Pulmonary symptoms are based descriptive reports of X-ray and CT scans. "Other" pulmonary symptoms include, but are not limited to, atelectasis, pleural scarring, pleural effusion, pulmonary edema, mild peribronchial thickening.
^^ non-severe include ER and In-patients only, severe includes patients in the ICU, on the ventilator or death.

BMI = body mass index, CVD = cardiovascular disease, CAD = coronary artery disease, CKD = chronic kidney disease, ESRD = end-stage renal disease, SOB = shortness of breath, COPD = chronic obstructive pulmonary disease
