# Supplementary material for: Predicting COVID-19 Severity with a Specific Nucleocapsid Antibody plus Disease Risk Factor Score
Source: mSphere. 2021 Apr 28;6(2):e00203-21. doi: 10.1128/mSphere.00203-21 (PMC8092137; doi:10.1128/mSphere.00203-21)
Supplement: TABLE S3 [file mSphere.00203-21-st003.pdf]

Oligo#	Sequence (5' to 3') with insertions denoted in lowercase and substitutions in bold	Product	Mutagenesis	Rounds
Oligo1	aggaagtggaggtggaggatccgggagctccagcCCGAGGGTGACGATCCCG	FlagTemplate	Q5	1
Oligo2	ttatcatcgtcatctttataatcaaccaatgcataGCCGAGGCGGAAAACATC			
Oligo3	attaaagtcgttcaccgtcgaaaaaggaatctatcagacctctaacGGTGGAGGATCCGGGAGC	Ep1	Q5	1
Oligo4	gtacactttgtttcactcagtggatctaatgcacaatcgaccgcatcTTTATCATCGTCATCTTTATAATCAACCAATGC			
Oligo5	gagcaagccttctaagcgctctttcattgaaGGTGGAGGATCCGGGAGC	Ep2	Q5	1
Oligo6	gggtcaggcaggatctgcgagaatccacttccTTTATCATCGTCATCTTTATAATCAACCAATGC			
Oligo7	tacacagttacctcccgcgtatacaaatGGTGGAGGATCCGGGAGC	Ep3	Q5	1
Oligo8	cgagttgtcaagttcacacatccacttccTTTATCATCGTCATCTTTATAATCAACCAATGC			
Oligo9	ttcttgcacgtgacgtatgtgcctgctGGTGGAGGATCCGGGAGC	Ep4	Q5	1
Oligo10	caccactccatgggcgctccacttccTTTATCATCGTCATCTTTATAATCAACCAATGC			
Oligo11	ggagctgaaaaaactgttggaacaatggaaccttgtaatcGGTGGAGGATCCGGGAGC	Ep5	Q5	1
Oligo12	tctacggtaatcgtaccgttcgagtccgccattccacttccTTTATCATCGTCATCTTTATAATCAACCAATGC			
Oligo13	tggagctgtgatcttacgcggacacctgcgtatcGGTGGAGGATCCGGGAGC	Ep6	Q5	1
Oligo14	atcactaattctgattccaacaggggtccacttccTTTATCATCGTCATCTTTATAATCAACCAATGC			
Oligo15	ttcgcacgcactcgctccatgtggtctGGTGGAGGATCCGGGAGC	Ep7	Q5	1
Oligo16	caagcggaagctcgcaattccacttccTTTATCATCGTCATCTTTATAATCAACCAATGC			
Oligo17	gcttcgtggttcactgcgcttacccagcacggaaagGGTGGAGGATCCGGGAGC	Ep8	Q5	1
Oligo18	tgtattattaggcagcccttgagggcgtccacttccTTTATCATCGTCATCTTTATAATCAACCAATGC			
Oligo19	tcaagggactaccttgcccaaggggttctatGGTGGAGGATCCGGGAGC	Ep9	Q5	1
Oligo20	ggtaattgtaacacgattgcagcgttattagcTCCACTTCCTTTATCATCGTCATCTTTATAATC			
Oligo21	gtaacccaagcgttcggtcgccgcgggGGTGGAGGATCCGGGAGC	Ep10	Q5	1
Oligo22	attatacgcttttgtagctccacttccTTTATCATCGTCATCTTTATAATCAACCAATGC			
Oligo23	gtctactctcgtgtaaaaaacttgaatGGTGGAGGATCCGGGAGC	Ep11	Q5	1
Oligo24	gtaaaaggaaggcttcactccacttccTTTATCATCGTCATCTTTATAATCAACCAATGC			
Oligo25	tttcaactttaatggcctgacggggaccggagtcctgactgaatccaatGGTGGAGGATCCGGGAGC	Ep12	Q5	3
Oligo26	ttgacacacttattttaaccaggtttgttgacttcttcggcccgcatacTTTATCATCGTCATCTTTATAATCAACCAATGC			
Oligo27	agacgctgttcgtgacccacagactctggagattttggacattacacctGGTGGAGGATCCGGGAGC			
Oligo28	gttgtatcggcgatgtcgcgtccaaattgctggaacggcagaaatttttATTGGATTCAGTCAGGACTCCG			
Oligo29	tgtctccgtcatcGGTGGAGGATCCGGGAGC			
Oligo30	cctccgaatgaacaAGGTGTAATGTCCAAAATCTCCAGAG			
Oligo31	ctgtatcaggatgtcaattgcacagaagtcGGTGGAGGATCCGGGAGC	Ep13	Q5	2
Oligo32	tacagcaacttggttacttgtgtttgtcccTTTATCATCGTCATCTTTATAATCAACCAATGC			
Oligo33	cccacttggcgcgtctacagcacaggcagtGGTGGAGGATCCGGGAGC			
Oligo34	cgtcagttggtcggcgtggatggctaccggGACTTCTGTGCAATTGACATCCTGATAC			
Oligo35	GCTGTACCAG**ggc**GTGAACTGTA	Ep13*	Q5	1
Oligo36	ACTGCCACCTGATTGCTG			
Oligo37	tatccacgtatcgggtacgaatggaacgGGTGGAGGATCCGGGAGC	Ep14	Q5	1
Oligo38	gcgtggaaccacgtcacatttccacttccTTTATCATCGTCATCTTTATAATCAACCAATGC			
Oligo39	tggagtgtctcccactaaattgaacgaccttGGTGGAGGATCCGGGAGC	Ep15	Q5	1
Oligo40	tagcatttgaaggttgagaacgatccacttccTTTATCATCGTCATCTTTATAATCAACCAATGC			
Oligo41	tcccccgacggagccgaagaaggataagaaaaaaaaaGGTGGAGGATCCGGGAGC	Ep16	Q5	2
Oligo42	aaggttttatacgcatcaatgtgtttatttccacttccTTTATCATCGTCATCTTTATAATCAACCAATGC			
Oligo43	acttccacagggaacgacactgccaaagggatttGGTGGAGGATCCGGGAGC			
Oligo44	tgcagtacagtggcagcattgttatttccacttccTTTATCATCGTCATCTTTATAATCAACCAATGC			
Oligo45	GGTGGAGGATCCGGGAGCTCCAGC	GA Vector	-	-
Oligo46	TTTATCATCGTCATCTTTATAATCAACCAATGCATAAGCCGAGGC			
Oligo47	CTGTATGTGGGCCCAAAAAGGGTGGAGGATCCGGGAGCTC	Ep17	GA	
Oligo48	GGCTGTACGCGTCCACTTCCTTTATCATCGTCATCTTTATAATCAACCAATGCATAAG			
Oligo49	ACGATGATAAAGGAAGTGGACGCGTACAGCCCACTGAAAG			
Oligo50	GAGCTCCCGGATCCTCCACCCTTTTTGGGCCCACATACAGTCG			
Oligo51	caaccgacaaatggtgtgggttaccagccgGGTGGAGGATCCGGGAGC	Ep18	Q5	1
Oligo52	gaaaccatatgactgcaagggaaaataacaTCCACTTCCTTTATCATCGTCATCTTTATAATC			
Oligo53	taataacttggattccaaagtaggaggcGGTGGAGGATCCGGGAGC	Ep19	Q5	1
Oligo54	gaattccaggcaataacacacccagtgaaTCCACTTCCTTTATCATCGTCATCTTTATAATC			
Oligo55	gaaatctaacttgaaaccgtttgaaaggGGTGGAGGATCCGGGAGC	Ep20	Q5	1
Oligo56	cggaaaagcctgtaaagatagttgtaattTCCACTTCCTTTATCATCGTCATCTTTATAATC			
Oligo57	cacgccttgcaatggggtagagggttttaatGGTGGAGGATCCGGGAGC	Ep21	Q5	1
Oligo58	gaccctgcctggtagatctccgttgagatgtcTCCACTTCCTTTATCATCGTCATCTTTATAATC			
Oligo59	caaccgacaaatggtgtgggttaccagccgGGTGGAGGATCCGGGAGC	Ep22	Q5	1
Oligo60	gaaaccatatgactgcaagggaaaataacaTCCACTTCCTTTATCATCGTCATCTTTATAATC			
Oligo61	acttccacagggaacgacactgccaaagggatttGGTGGAGGATCCGGGAGC	sEp9	Q5	1
Oligo62	tgcagtacagtggcagcattgttatttccacttccTTTATCATCGTCATCTTTATAATCAACCAATGC			
Oligo63	tccgggtacaaagttaccaaagaacttccacGGTGGAGGATCCGGGAGC	mEp9	Q5	1
Oligo64	gcaaattgagtaactatcgctgaatcattgttTCCACTTCCTTTATCATCGTCATCTTTATAATC			
Oligo65	tcccggaactatttaccccaaggatactatGGTGGAGGATCCGGGAGC	hEp9	Q5	1
Oligo66	gggaatctagtgggaatcgcctcctgagtagtTCCACTTCCTTTATCATCGTCATCTTTATAATC			
Oligo67	agtattgccttgccacctgagttatctGGTGGAGGATCCGGGAGC	nEp9	Q5	1
Oligo68	aaatttcggttcaagcggcttttgattTCCACTTCCTTTATCATCGTCATCTTTATAATC			
Oligo69	TTTTGCGCCGACATCATAACGGTTCT	pm1165a P8	-	-
Oligo70	TATGGGGTTTTGCTAAACAACTTTCAACAG			
Oligo71	agatgacgatgataaaggaagtggataataaGGCGGTTCGGCAGGCGAA	eGFP-FLAG	Q5	1
Oligo72	ttataatcgctggagctcccggatcctccaccTTTGTATAGTTCATCCATGCCATGTGTAATCCC			
Oligo73	tcaagggactaccttgcccaaggggttctatAGCGGAAGTGGAGATTATAAAGATGAC	eGFP-Ep9	Q5	1
Oligo74	ggtaattgtaacacgattgcagcgttattagcGGAGCTCCCGGATCCTCC			
Oligo75	TAATACGACTCACTATAGGG	T7	-	-
Oligo76	GCTAGTTATTGCTCAGCGG	T7 term	-	-

List of oligos used in Q5 site-directed mutagenesis (Q5) or Gibson Assembly (GA) for the cloning of putative epitopes Ep1-22 for phage display or eGFP recombinant protein expression. For epitope constructs between 100-210 bp in length, multiple rounds of Q5 were denoted.
